# Scaling Urban Methane Emissions: Utility of Single-Site Measurements in Five Urban Domains

**DOI:** 10.1021/acs.est.5c03844

**Published:** 2025-07-09

**Authors:** Kimberly L. Mueller, Anna Karion, Israel Lopez-Coto, Julia Marrs, Vineet Yadav, Genevieve Plant, Joseph Pitt, Zachary R. Barkley, James Whetstone

**Affiliations:** † Special Programs Office, 10833 National Institute of Standards and Technology, Gaithersburg, Maryland 20899, United States; ‡ 53411 Jet Propulsion Laboratory, Pasadena, California 91109, United States; § Climate and Space Sciences and Engineering, 1259 University of Michigan, Ann Arbor, Michigan 48109, United States; ∥ School of Chemistry, University of Bristol, Bristol BS8 1TS, U.K.; ⊥ Department of Meteorology and Atmospheric Science, The Pennsylvania State University, State College, Pennsylvania 16802, United States

**Keywords:** methane, urban, emissions, northeast, greenhouse gas, in situ observations

## Abstract

Urban methane (CH_4_) missions remain poorly understood due to limited observational constraints. Most estimates rely on bottom-up inventories based on assumed emission factors and activity data or downscaling methods, which often underestimate emissions, sometimes by a factor of 2 or more in United States and European cities. While satellite and mobile observations can improve understanding, they face limitations in spatial resolution, coverage, and frequency. In contrast, fixed in situ measurements calibrated to World Meteorological Organization standards offer high precision continuous data, although with limited spatial coverage due to logistical constraints. This study uses in situ observations from single tower sites in five northeastern United States cities to estimate total urban CH_4_ emissions using a Bayesian scaling factor framework. Despite limited spatial sampling, the approach yields robust emission estimates consistent with other studies. To explore drivers of variability, the analysis examines correlations between inferred emissions and urban characteristics including population, residential gas usage, and infrastructure. Results show that residential building volume outperforms population as a predictor in some regions, highlighting the importance of infrastructure-specific factors. By demonstrating a scalable observation-based approach using minimal sites, this work addresses key gaps in urban CH_4_ monitoring and emphasizes the value of robust measurements and tailored proxies for improving emission estimates in diverse urban settings.

## Graphical abstract

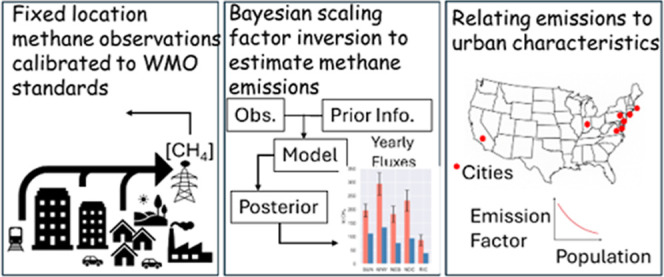

## 1 Introduction

Methane (CH_4_) emissions from urban areas remain poorly understood. Most of our current understanding relies on inventory-based approaches that generally use emission factors and activity data, often along with downscaling methods, which may be highly uncertain or involve assumption.^1–4^ These assumptions and non-accounted sources, such as fugitive emissions from leaky infrastructure, can lead to significant uncertainties and underestimations, as demonstrated by numerous urban studies that have estimated CH_4_ emissions exceeding inventory-based estimates by a factor of 2 to three in U.S. and European cities.^5–10^

Satellite retrievals have been used to improve urban CH_4_ emission estimates.^10–15^ However, the use of these measurements can face several limitations. For example, satellite-based estimates, while offering broad spatial coverage, often lack the resolution needed to capture urban-scale variability in both space and time. Their effectiveness is further constrained by a limited number of valid observations due to persistent cloud cover, aerosols, and other atmospheric complications.^16^ In addition, many satellites have detection thresholds that limit their ability to detect the relatively diffuse and spatially distributed emissions characteristic of urban areas. For instance, instruments such as GHGsat have a limit of detection on the order of 1 tonne per hour for point sources, making them less sensitive to smaller, distributed urban sources.^17^ Many regions, particularly in Africa and South America, remain poorly observed by space-based instruments due to some of these challenges.

To enhance the accuracy of satellite observations, global mapper CH_4_ measurements (e.g., from the TROPOspheric Monitoring Instrument, TROPOMI)^18^ are validated against ground-based networks such as the Total Carbon Column Observing Network (TCCON) and Collaborative Carbon Column Observing Network (COCCON), which act as transfer standards aligned with World Meteorological Organization (WMO) calibration protocols. Detailed analyses are conducted over these validation sites to develop bias correction schemes for satellite data. Nevertheless, a key weakness of this approach is the sparse distribution of ground-based sites in the Southern Hemisphere, which, along with potential biases related to surface albedo, can limit the accuracy of satellite-derived estimates.

In addition to satellite-based methods, observations of atmospheric CH_4_ from moving platforms, such as aircraft-based in situ measurements, are also used to refine urban CH_4_ emission estimates.^15,19–22^ These measurements, which can be directly calibrated to WMO standards, circumvent many of the issues inherent to satellite retrievals. Despite their value, such observations typically provide only snapshot estimates from specific field campaigns, lacking the temporal continuity necessary to fully capture the dynamic nature of urban CH_4_ emissions over time.^23^

Finally, in situ CH_4_ measurements from fixed locations provide continuous, high-precision data calibrated to WMO standards, making them directly comparable across sites and regions—similar to any observations calibrated to these standards.^24^ However, in situ measurements from permanent sites are spatially limited due to the high costs of equipment, operation, and the logistical challenges of site selection^20,25^ and maintenance. Despite their spatial limitations, these measurements can be invaluable in understanding CH_4_ emissions when integrated with advanced modeling approaches.^26,27^ Moreover, dense networks of in situ towers, such as the National Institute of Standard and Technology (NIST) testbeds, offer a way to overcome spatial constraints, providing rich data sets that can capture variability of GHG emissions within urban domains.^28–30^ Such networks can also serve as benchmarks for validating satellite-based CH_4_ emissions, improving confidence in broader-scale estimates.

This study presents an approach to estimate urban CH_4_ emissions using in situ observations from a single fixed location in an urban domain. Employing a Bayesian scaling factor framework,^31,32^ the method leverages the continuous temporal coverage and high precision of fixed in situ observations to provide estimates of total urban CH_4_ emissions. Although this method does not resolve spatial variability, the approach offers a straightforward and replicable way to infer emissions for entire urban domains. This method, like others, can be evaluated and validated in regions with dense site networks, such as the NIST testbeds, before being applied to cities with fewer observational resources.

Beyond estimating CH_4_ emissions, this study also investigates the relationship between estimated emissions and proxy variables related to urban characteristics, including infrastructure, demographics, and waste. Understanding these relationships is critical for identifying the drivers of urban CH_4_ emissions and for developing targeted mitigation. Proxy variables help link the form and function of urban areas to their emission profiles, providing insights that can inform both mitigation pathways and future research.

By pioneering a single-site approach and investigating the utility of proxy variables, this study aims to fill key gaps in urban CH_4_ monitoring. Importantly, this approach demonstrates the potential for significant cost reduction by leveraging a limited number of fixed-site locations. This not only enhances the economic feasibility of long-term urban CH_4_ monitoring but also enables the collection of high-quality data traceable to WMO standards for assessing emission trends and magnitudes. Furthermore, having one or more well-instrumented sites provides a framework for integrating other types of CH_4_ measurements, such as satellite observations^33,34^ and in situ observations from mobile platforms^35^ or lower-cost/medium-precision sensors.^36–38^ This integrated approach can significantly improve atmospheric constraints on emissions, enabling more targeted source identification and ultimately supporting efforts to address urban CH_4_ contribution to the atmospheric burden of GHGs.

## 2 Methods

### 2.1 Observations, Analysis Domain, Meteorology, and Dispersion

In the Northeastern U.S., there are numerous in situ CH_4_ observation sites in and around urban areas, as defined by the U.S. Census,^39^ that can help us better understand urban CH_4_ dynamics across different demographic settings. This area is part of the National Institute of Standards and Technology (NIST) Northeast Corridor (NEC) testbed^40^ which extends to western Illinois, southern Canada, and Georgia (Figure 1f). Our study leveraged high-precision, hourly averaged CH_4_ observations from five urban atmospheric monitoring sites situated along the northeastern seaboard (Figure 1f) of the NEC testbed.

**Figure 1:**
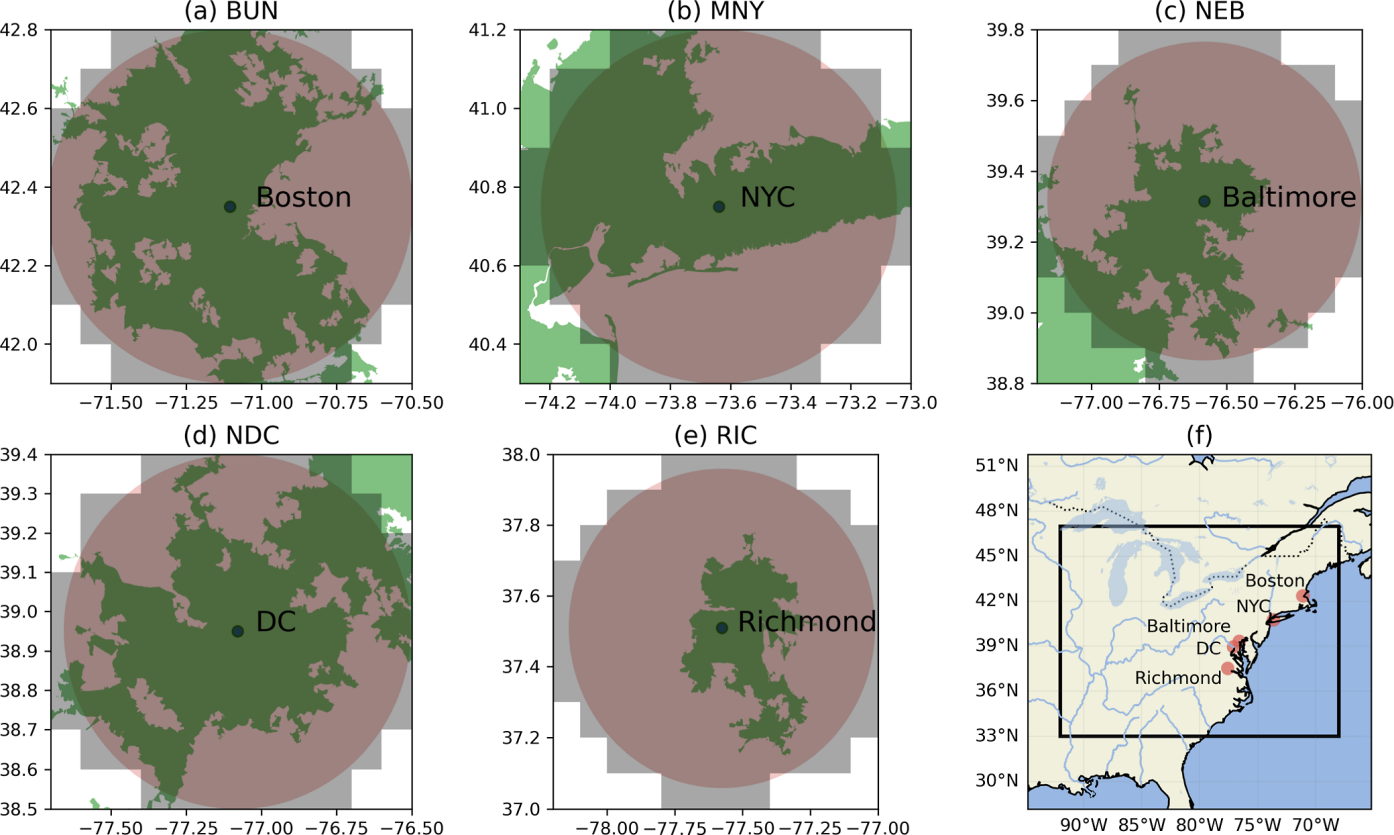
(a–e) Observational site locations (black dots; three letter site code above plots that is an abbreviated observational site name, full names are provided in Table S1 and denoted later in the text) whose associated observations are used in the analysis. Census-designated urban areas are shown in green while 0.1 degree mask (gray areas) is our estimation domain for each urban site, defined as a circle with a 50 km radius (red areas). (f) The regional extent of our analysis (“d01” domain) where the locations of the red circles correspond to those in (a–e).

Four of the five sites are part of the NIST NEC project, while the fifth is operated by Boston University (BUN)^41^ (Figure 1a–e). The afternoon hourly observations used in this work largely span January 2017 through December 2021 (SI-1 and Figure S1). We used hourly mole fractions from January 2017 to May 2020 from the BUN site given the limited availability of data over the study period. Details on the locations are provided in Table S1.

Other studies have shown that mole-fraction variability observed at fixed locations is most sensitive to local emissions.^42^ Our “sensitivities” to surface emissions are determined by releasing ∼1000 particles from the location of each measurement site and tracking these particles back in time and space for 120 h using the Stochastic Time-Inverted Lagrangian Model (STILT)^43^ coupled to two meteorological models.^43^ We used a NIST custom-configured Weather Research and Forecasting (WRF) model that originated from the National Center for Atmospheric Research (NCAR) and the European Centre for Medium-Range Weather Forecasts (ECMWF) Reanalysis v5 product (ERA5) for the study.^44,45^ Both products were also described in Karion et al.^26^ and more details are provided in SI-2. We refer to the sensitivity of an observation to all emissions in time and space as an observational footprint that makes up a Jacobian matrix (**H**). There are 33,600 0.1 degree grid cells in d01, so the dimension of **H** is large as will be discussed in Section 3.

To identify the area most influenced by local emissions, we plotted the decay of the sum of sensitivities (from both WRF and ERA5) for afternoon hourly observations as a distance from the observation location (Figure S2). The plot shows that the sensitivity of the measurements generally plateaus after approximately 50 km. Guided by this knowledge, we delineated our analysis areas by aggregating 0.1 degree grid cells that predominantly encompass a 50 km radius centered on a site’s location (Figure 1a–e).

The footprints derived from each meteorological model vary in both spatial patterns and overall magnitudes; for example, the footprints based on ERA5 data are generally stronger (i.e., have larger magnitudes) than those produced by the WRF model (see Figures S3–S5). Moreover, while some sites exhibit seasonal sensitivity, often with higher values in winter compared to summer, others do not show a clear seasonal trend. The impact of these sensitivities varies across estimation domains because each meteorological model represents atmospheric parameters (such as wind and temperature, and planetary boundary layer) differently.

### 2.2 Study Area and Analysis Setup

Figure 2 illustrates the different components considered in this study. CH_4_ mole-fraction observations from each site are influenced by (1) inner domain local anthropogenic emissions (**s**_p,in_), (2) outer domain (d01) anthropogenic emissions (**s**_p,out_), which exclude those sources inside the inner estimation domain (3) wetland emissions from the entire regional domain (**s**_d01,wet_), and (4) background emissions originating outside d01 that are advected into the domain (**BG_gb_**) (Figure 2). The subscript “p” denotes that the emissions inside and outside the estimation domain that have not been adjusted using estimated scaling factors from the Bayesian inversions.

**Figure 2:**
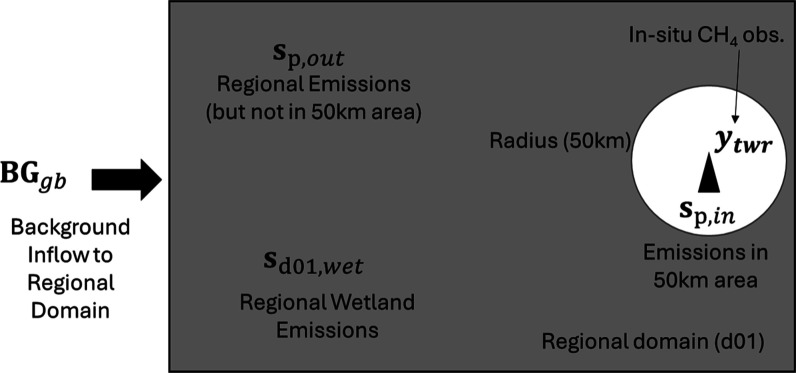
Conceptual diagram which shows the local area (50 km radius circle) for which we quantify and investigate the nature of CH_4_ emissions. To do so, we use atmospheric observations (***y*_twr_**) measured at a site (black triangle). The full extent of the of analysis (gray rectangle) is the d01 regional domain and the local areas around each site. We also use regional d01 emissions (**s**_p,out_), emission within a 50 km radius around the observational location (**s**_p,in_), wetland emissions (**s**_d01,wet_), and background mole fractions flowing into our d01 domain (**BG_gb_**). Note that we ran individual inversion for each of the five locations in Figure 1 using the observations as measured from the specific measurement site.

Using the Jacobian (**H**), we can write the mole fraction observed at a given location and time (**y_twr_**) as the sum of each of the three emission contributions as observational components while also including **BG_gb_** and the error term (**ε**) as shown

1 $$y_{twr}=Hs_{p,in}+Hs_{p,out}+Hs_{d01,wet}+{BG}_{gb}+\varepsilon$$

Note that our error term, (**ε**) is primarily the sum of errors in (1) measurement **ε_measurement_**, (2) atmospheric-dispersion meteorological modeling **ε_transport_**, and (3) background **ε_BGd01_**, among others.^46,47^ These components are discussed further in SI-5.

### 2.3 Gridded Methane Inventories and Wetlands

We used a suite of anthropogenic CH_4_ emission products at 0.1 degrees, which cover our d01 domain, to convolve with our Jacobian matrix **H** at each observational time to generate our modeled enhancements (**Hs**_p,in_ and **Hs**_p,out_ in eq 1).

We used two publicly available, research grade inventories, one generated by the US Environmental Protection Agency (EPA; Figure S6)^1^ and the other from the European Commission (Emissions Database for Global Atmospheric Research or EDGAR^48^ version 8, referred to as ED8 henceforth; Figure S7). For each year of our analysis, we used the corresponding emission product except for 2021 for the EPA inventory. At the time of this analysis, the EPA had not published 2021 CH_4_ emissions, so we used the 2020 emissions instead. The native spatial resolution for each of these emissions products is 0.1 degrees. There is almost no interannual or monthly trends in these emission products. Therefore, much of the variability in the modeled enhancements are the result of the variability in the atmospheric transport and dispersion models as noted earlier.

Additionally, we have emission inventories that were created by other CH_4_ studies^26,31,41^ specifically for their domain and year of interest at finer resolutions than 0.1 degrees. These include areas around Baltimore (NEB)^26^ and Washington, D.C. (NDC)^26^ as well as for Boston (BUN)^41^ and New York City (MNY)^49^ (Figure S8; locations shown in Figure 1a–e). These custom inventories incorporated activity data and emission factors from sources selected specifically for their study domain. Therefore, we expect these CH_4_ products to be more representative, in magnitude and spatial distribution, of the actual CH_4_ emissions even though most of these studies concluded that the magnitude of the custom inventories is too small when compared to observations used in inversions or scaling methods. More information on the inventories is provided in SI-3.

Each custom inventory was aggregated to 0.1 degrees to ensure spatial consistency with those from the EPA and ED8. None of these custom inventories have any temporal variability. We embedded each custom inventory into both the regional EPA and ED8 products since the latter products have different regional spatial variability. Thus, we have four inventory ensemble members, EPA, ED8, EmbEPA, and EmbED8, where the last two refer to the versions with embedded custom inventories. Note, the spatial distribution of these products both within our local areas (**s**_p,in_) and regional domain (**s**_p,out_) (Figure S9) can vary significantly. The modeled enhancements (**Hs**_p,in_ and **Hs**_p,out_) also vary in magnitudes (Figure S10).

Wetlands are a major global source of CH_4_ and can significantly influence atmospheric observations. In our analysis, we used a custom wetland emissions model for the d01 domain, developed using a method similar to Karion et al.,^26^ and treated these emissions as if they were perfectly known. A recent inverse modeling study highlight the challenges of attributing emissions across heterogeneous landscapes and at different spatial scales.^50^ The broad agreement between our modeled fluxes and their regional estimates in the overlapping area within our d01 domain provides some confidence in the plausibility of our wetland emission values, despite differences in methodology and resolution.

While our model likely provides a reasonable estimate of wetland-related CH_4_ enhancements, the true wetland emissions are inherently uncertain. To account for this and other sources of uncertainty, we included an additional error term in the model-data mismatch covariance matrix (**R**; see SI-5 for more information). However, large or systematic biases in wetland emissions, particularly if they vary seasonally or spatially, may not be fully captured by this generalized uncertainty term. As a result, this simplification represents a known limitation of our inversion system and may affect the accuracy of anthropogenic CH_4_ estimates, particularly in regions where wetland contributions are significant.

To further probe how much of an impact wetlands could have on our estimates compared to anthropogenic emissions from urban land-use types, we convolved the National Land Cover Database (NLCD, 2016)^51^ with our Jacobian matrix **H** (Figure S11). The relative percentage of “wetland” land-use categories when directly compared to “urban” is not significant except in the 50 km radius around RIC. Thus, although there may be biases, we expect that the dominant portion of **Hs**_p,in_ is from the emissions from the NLCD “urban” domains within the 50 km. Note that we also evaluated other wetland models such as various ensemble members within WetCharts^52^ to account for seasonality, but they had minimal impact on our results.

### 2.4 Global Background

To estimate emissions for a specific 50 km radius, we need to account for CH_4_ emissions originating outside the area and advected into the study domain. For BG_gb_, we used two global model transport models as ensemble members, the European Global Copernicus Atmosphere Monitoring Service^53^ (aka, CAMS, CAMS17r1, see SI-4 for more information) and NOAA’s Carbon Tracker Methane^54^ (CT-CH4), that provides CH_4_ mole fraction fields which we sample at the edge of our regional d01 domain (Figure 2). We used the 4-D end points (i.e., *x*, *y*, *z*, *t*) of particles, that were released using STILT to make **H**, to sample these global CH_4_ model(s). We then averaged the CH_4_ mole fractions from these end points to generate a BG_gb_ for each observation and then repeat the exercise for all the other observed CH_4_ mole fractions at sites to get **BG_gb_** used in the analysis.

We assumed that the uncertainties associated with CH_4_ **BG_gb_** may have a significant impact on our estimated emissions comparable to those associated with **Hs**_p,out_ along with wetland emissions (**Hs**_d01,wet_).^22^ One main reason is related to the absolute magnitude of the global values of CH_4_ compared to both **Hs**_p,out,_ **Hs**_p,in_, and **Hs**_d01,wet_. A small percentage error in the global background value can significantly impact our ability to ascertain the portion of the observed mole fraction from local emissions. It has been shown for carbon dioxide (CO_2_) that the choice of boundary conditions have had significant impact on estimated CO_2_ emissions in continental inversions.^55^ Given that CH_4_ emissions are more uncertain than those of CO_2_, we expect that any errors **BG_gb_** could have even larger impacts for our small domain.

To address the influence of background methane concentrations (**BG_gb_**) on our emissions estimates, we performed a comprehensive evaluation of global model products as described in the Supporting Information (SI-4). We compared several CAMS ensemble members and CT-CH_4_ to NOAA aircraft profiles near the regional domain (d01) boundary to assess their fidelity. Due to limitations in aircraft coverage, we supplemented this with a statistical regression analysis that incorporated in situ observations from regional towers, convolved emissions, and transport model outputs. This analysis identified one CAMS product (CAMSv17r1s) as the most consistent ensemble member, but also revealed a systematic positive bias. We estimated an annual offset (BG_adj_) of 10.2 ppb using particle trajectory sampling and aircraft data and validated this with multilinear regression residuals. This offset was applied to (**BG_gb_**) values in our inversion to mitigate background-driven biases in the emission estimates. While this adjustment approach is relatively complex, it reflects the significant role of background mole fractions in regional CH_4_ inversions and provides a framework for similar applications elsewhere.

However, we also recognize that the analysis in S1-3 may be too complicated for practical implementation in future or operational studies. A simpler initial assessment may involve comparing aircraft observations with 4-D mole fractions from a global model if measurements are available. Regardless, the evaluation of 4-dimensional CH_4_ products from global models before using them as backgrounds for most emission estimation methods is essential.

## 3 Bayesian Scaling Factor Inversion Framework

Similarly to Pitt et al.,^31^ we employed a simple Bayesian inversion system to estimate scaling factors on modeled CH_4_ enhancements from the 50 km radius around each urban site and the regional domain, as well as a scaling factor on the adjusted global background (**BG_gb_**–BG_adj_), using afternoon hourly atmospheric observations.

The inversion process was performed every 8 days, with a 4 day overlap between consecutive inversions. This approach ensures continuity in the estimated scaling factors. Importantly, scaling factors are assumed to be constant within each 8 day inversion window. We assumed λ_p,1_, λ_p,2_, λ_p,3_ that associated with **Hs**_p,in_, **Hs**_p,out_, and (**BG_gb_**–BG_adj_) respectively, can be applied to emissions themselves to provide CH_4_ estimates in both domains. We do not estimate a scaling factor for wetlands emissions (**s**_d01,wet_) since we assume that these emissions are perfectly well-known.

The dimension of **Hs**_p,in_ and **Hs**_p,out_ is *N* × 1 where *N* is the number of observations in each inversion. Since we assume no temporal variation in **s**_p,in_ and **s**_p,out_, we sum the **H** matrix corresponding to a given observation time with those from all other observations within the same inversion period. As a result, the number of columns in **H** remain fixed at 33,600, while the number of rows correspond to the number of observations used in the inversion.

The following likelihood equation was minimized to estimate the most likely scaling factors ( λ̂ ) and their associated uncertainties ( $V_{\mathrm{\lambda ̂}}$ ). Note that **λ̂** is a (3 × 1) vector for each inversion iteration. The degrees of freedom is the number of observations (∼40 but depends on the time of year, filter requirements, and data gaps explained in SI-6) used in each inversion minus three.

The likelihood equation is:

$$L_{\lambda }={\left(y_{twr}-\lambda_{1}Hs_{p,in}-\lambda_{2}Hs_{p,out}-Hs_{wet}-\lambda_{3}\left({BG}_{gb}-{BG}_{adj}\right)\right)}^{T}\times R^{-1}\left(y_{twr}-\lambda_{1}Hs_{p,in}-\lambda_{2}Hs_{p,out}-Hs_{wet}-\lambda_{3}\left({BG}_{gb}-{BG}_{adj}\right)\right)+{\left(\lambda_{p}-\lambda \right)}^{T}Q^{-1}\left(\lambda_{p}-\lambda \right)$$ 2

When minimized yields estimated scaling factors and their uncertainties:

3 $$\mathrm{\lambda ̂}=\lambda_{p}+QH^{T}{\left(HQH^{T}+R\right)}^{-1}\left(y-H\lambda_{p}\right)$$

4 $$V_{\mathrm{\lambda ̂}}=Q-QH^{T}{\left(HQH^{T}+R\right)}^{-1}HQ$$

Such that:

5 $${ŷ}_{twr}={\mathrm{\lambda ̂}}_{1}Hs_{p,in}+{\mathrm{\lambda ̂}}_{2}Hs_{p,out}+Hs_{wet}+{\mathrm{\lambda ̂}}_{3}\left({BG}_{gb}-{BG}_{adj}\right)$$

with resulting estimated emissions and associated uncertainties:

6 $${ŝ}_{in}=s_{p,in}\times {\mathrm{\lambda ̂}}_{1}$$

7 $$V_{{ŝ}_{in}}={\left(V_{\mathrm{\lambda ̂}}H\right)}_{1}$$

Note that weighting between the observations and the prior is determined by model data mismatch covariance matrix (**R**) and the prior error covariance matrix (**Q**). SI-5 describes how these matrices were configured for our analysis.

We ran five separate inversions, one for each 50 km radius. Critically, the inversion associated with each site uses only the observations specific to that site; no data from other sites are included. For each individual inversion, we generated 16 ensemble members from the combination of four inventories, two meteorologies, and two background fields. We maintained a consistent configuration for the inversion (including error estimation and filtering) across all five inversions. This approach ensured that observed differences in emission estimates are attributable to actual variations between areas, rather than artifacts of inconsistent methods, data, or assumptions.

SI-6 provides histograms of ${\mathrm{\lambda ̂}}_{1}$ for **s**_p,in_ and ${\mathrm{\lambda ̂}}_{3}$ for **s**_p,out_ (Figure S17). SI-6 also gives details on filtering criteria (e.g., ensuring there are enough observations within an 8 day period to estimate methane emissions, etc.) and inversion metrics from our inversion runs (Figures S18 and S19), showing that the inversions performed well, and the estimated scaling factors are largely independent of one another (Figure S20). This independence is further evidence that we can distinguish our 50 km radius estimates from those associated with d01.

To further justify our choice of the estimation domain, we compared the variability of **Hs**_p,in_ and **Hs**_p,out_. For this comparison, we computed the relative difference between the standard deviation of enhancements from (1) local **Hs**_p,in_ and (2) regional emissions **Hs**_p,out_, normalized by their respective means (eq S-23). The results indicate **Hs**_p,in_ exhibit greater relative variability than **Hs**_p,out_, with an average slightly greater than 50%. This suggests that the difference in variability helps us isolate ${\mathrm{\lambda ̂}}_{1}$ from ${\mathrm{\lambda ̂}}_{2}$ . Results are summarized in Table S3.

## 4 Emission Results

Mean emission estimates exhibit little interannual variability (Figure 3). While our methodology, domain extent, observational data, and uncertainty quantification preclude the detection of a significant declining trend as suggested by previous studies,^26^ our estimates are statistically significantly higher than inventory values (1σ) for all regions except BUN, even when compared to domain-specific inventories (Emb-EPA, Emb-ED8). Furthermore, the magnitude of our estimates aligns with those reported in the literature, illustrating the capability of our simple method to accurately estimate city-wide annual emissions. To further validate our findings, CH_4_ emissions were quantified within 50 km radius using observations from closely located sites in Boston (BUN and COP), Baltimore (NWB and NEB), and Washington, D.C. (NDC and ARL) (Table S1). These 50 km radius estimates, while not shown, were like those obtained using only the BUN, NEB, and NDC observations for 2017, albeit with expected reduced uncertainty, further supporting our methodology.

**Figure 3:**
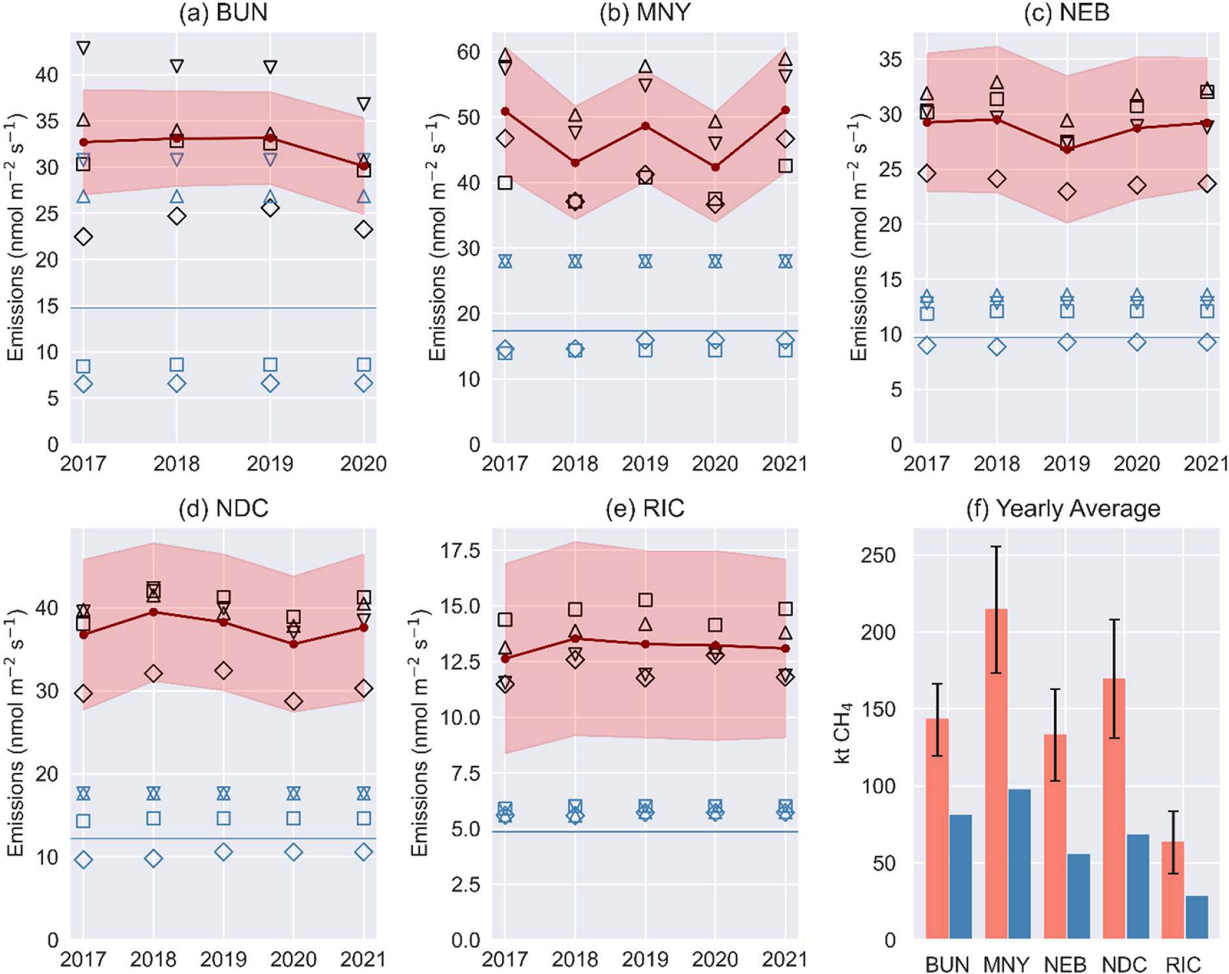
(a–e) Annual CH_4_ estimates for regional areas around the sites with 1σ uncertainties represented by red shading. Estimates are the average of those using different inventories for both WRF2 and ERA5. The black symbols are the individual ensemble estimates (Emb-ED8 downward triangle, Emb-EPA upward triangle, ED8 squares, and EPA diamonds). The blue symbols represent the inventories where the symbols correspond to those of the estimates. (f) Average annual estimated emissions and uncertainties at 2σ (orange bars) and nonscaled annual emissions averaged across all emission products in kilo tonnes (kt) (blue bars).

To contextualize the underestimation evident in Figure 3, the average annual difference between our estimated emissions (Figure 3a–e, red line) and inventory-based values (Figure 3a–e, blue line) was quantified. This discrepancy averaged 80 kt ±31 kt CH_4_ (2.24 MtCO2e ± 0.87 MtCO2e), ranging from 35 kt ± 20 kt CH_4_ (0.98MtCO2e ± 0.56 MtCO2e) in RIC to 117 kt ± 41 kt CH_4_ (3.28 MtCO2e ± 1.15 MtCO2e) in MNY using a global warming potential (GWP) of 28.^56^ While seemingly minor compared to the 7,857 kt CH_4_ (220 MtCO2e) from the U.S. natural gas sector in 2022,^57^ this underestimation becomes significant when considering the approximately 2650 urban areas within the U.S.^58^ This highlights the substantial, albeit often overlooked, contribution of urban CH_4_ emissions at the national scale.

The comparison of our seasonal CH_4_ emission estimates to those from Karion et al.^26^ and Pitt et al.^31^ provides an essential validation of our method and results (Figure 4). Despite the presence of large 2-σ uncertainties, the alignment of our mean seasonal emissions (Figure 4, red line) with these independently published magnitudes and trends underscores the robustness of our approach. Note, we only use monthly averaged points if there are three valid estimates within the month (Figure S21) which can lead to significant gaps in the timeseries. A comparative analysis of winter (November–January) and summer (June–August) emissions indicated a less pronounced seasonal cycle compared to Karion et al.^26^ (Figure 4f), perhaps due to gaps or other observational constraints. Regardless, our comparative analysis of seasonal emissions highlights nuanced regional variations in the seasonal cycle, such as the pronounced seasonality observed in New York City (MNY) versus the minimal variability in the Richmond (RIC) region. These findings not only reinforce the validity of our approach but also contribute to a more detailed understanding of regional CH_4_ emissions, in different estimation areas, revealing spatial heterogeneities.

**Figure 4:**
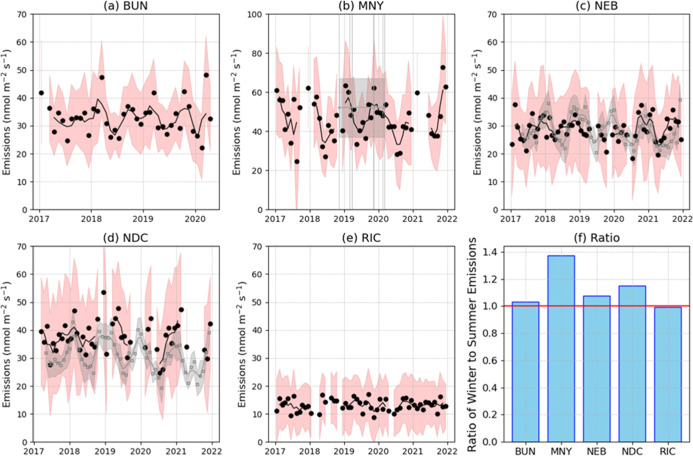
(a–e) Black circles are monthly emission averages. Only months that have three or more 8 day estimates are shown. The black lines are 3 month running means and red shadings are 2σ uncertainty levels estimated analytically from the inversion. Gray circles on (c,d) are monthly means from Karion et al.^26^ along with associated uncertainties, gray shading, based on ensemble spread used in cited work. The gray lines are the 3 month moving means. The horizontal gray line on (b) is the estimate from Pitt et al.^31^ with uncertainties from an ensemble spread shown in gray shading. Pitt et al.^31^ used 9 different flight days between 2018 and 2020. These individual flight times are shown as vertical gray lines. The ratios (f) are the monthly average wintertime (November–January) emissions divided by the average summertime monthly emissions (June–August). The red line indicates a ratio of one, i.e., no seasonality.

## 5 Characteristic Analysis

Having confidence in our emission estimates, we analyzed ancillary data representing human activities linked to urban CH_4_ emissions to explore relationships that may explain variations across urban areas. These data include 13 proxies for major emission sources, such as waste acceptance rates for landfill emissions, residential natural gas use for residential emissions, and pipeline infrastructure for natural gas distribution emissions (see SI-7 and Table S4).

Much of the proxy data was compiled at fine spatial scales, such as from the U.S. Census block level (Figure S22) or at the Local Distribution Company (LDC) scales for natural gas. We downscaled such data to the 0.1 degree scale using spatial proxies like the length of roads from OpenStreetMaps, and the total volume of residential houses or commercial buildings derived from the Homeland Infrastructure Foundation-Level Data (HIFLD),^59^ and then reaggregated to our 50 km radii. Therefore, all proxy data is consistent across all the five 50 km domains (see Section SI-8 for more details).

High positive correlations between most activity data (Figure S23), particularly those related to natural gas use, highlight their strong interdependence. In contrast, the negative correlation between waste acceptance rates and emissions may reflect (1) improved landfill management in larger metropolitan areas or (2) where waste generated in an urban area ultimately resides. Highly urbanized cities like New York City ship their waste elsewhere while there are large active landfills near Richmond’s urban domain. However, the limitations in the proxy or underlying nonlinear relationships warrant further investigation.

As shown in Figure 5, population exhibits a strong correlation (0.9) with CH_4_ emissions, as expected, given the link between larger populations and increased energy demands. However, residential volume shows an even stronger correlation (0.98), suggesting it may serve as a better predictor of CH_4_ emissions, especially in older urban areas with aging infrastructure that may contribute to higher leakage rates. These findings highlight the potential for residential volume to capture urban-specific characteristics influencing emissions more effectively than population alone.

**Figure 5:**
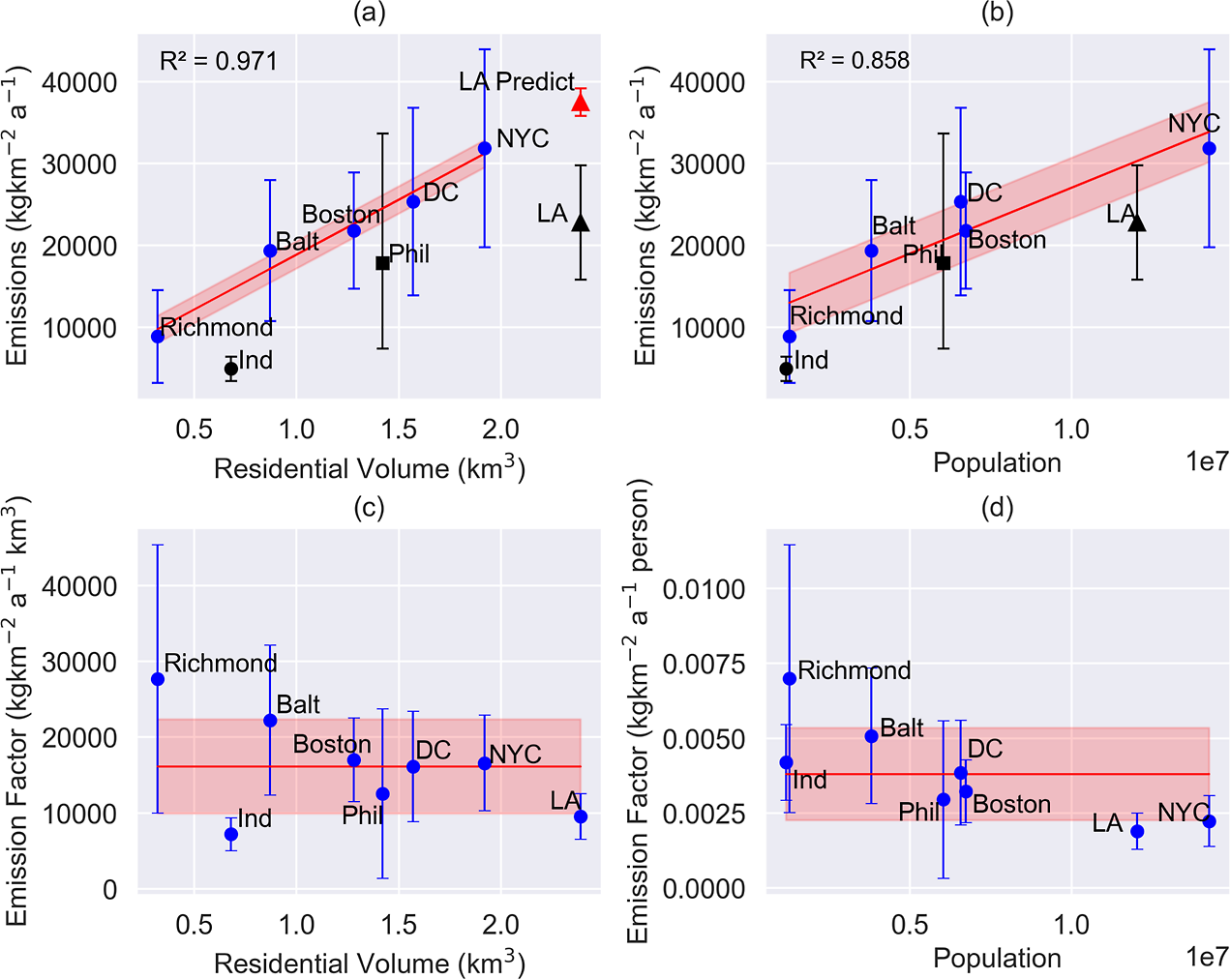
Blue circles and error bars (2σ) are average estimates (across the entire analysis time for all members) from this study plotted against both residential volume (a) and population (b); red line is the linear fit to these five estimates with uncertainty associated with the slope in red shading. Estimates from Yadav et al.^9^ (Los Angeles, triangle) Plant et al.^8^ (Philadelphia, squares), and Indianapolis (circle, Barkley, personal communication) are plotted against both residential volume and population to show how well predicted values, using activity data from these regions and the slopes, compare to reported values. For (a) Los Angeles predicted value is shown in red triangle. Emission factors (c and d), estimated by emissions for all eight urban emissions and uncertainties (blue dot and bars) divided by either population and residential volume with the mean shown as a red bar and uncertainty around the mean as red shading.

To refine these insights, we conducted two simple linear regression analyses using CH_4_ emissions, from our five estimation domains, as the dependent variable and either population or residential volume as independent variables (Figure 5a,b). Models using residential volume had smaller uncertainties in slope estimates, suggesting improved predictive performance. However, independent comparisons with CH_4_ estimates from Los Angeles,^9^ Philadelphia,^8^ and Indianapolis (Barkley, personal communication) revealed regional differences. While population may serve as a reasonable proxy in cities where emissions are driven primarily by consumption-related leaks, it may be less applicable in cities like Richmond if other sources dominate or if emissions are more constant over time. This highlights the limitation of using a single proxy (such as population) across all cities.

Finally, we investigated the relationship between emissions and these two proxies (Figure 5c,d). For these plots, emission estimates for all eight urban domains (five from this work and three from previous work as indicated in the caption) are divided by population or residential volume to obtain emission factors (EFs). We calculate the mean and uncertainty of these EFs. The EFs are not uniform or constant across all the cities, illustrating a potential nonlinear relationship between emissions and proxies, and suggesting larger urban areas may exhibit efficiencies that complicate linear assumptions. For example, larger urban areas may emit less CH_4_ per capita due to efficiencies in infrastructure or management. This scaling behavior has also been observed in studies of urban CO_2_ emissions where per capita emissions tend to decrease with increasing city size.^60^ While these findings provide valuable insights, the limited sample size of eight urban domains underscores the need for further analysis with a larger data set to establish more robust and generalizable relationships.

## 6 Discussion of Main Findings

The methods presented in this study, applied separately for each of our five urban estimation domains, demonstrate the ability to estimate annual CH_4_ emission and partially capture seasonal patterns using observations from a single in situ observation site. Our estimates compare well with emissions estimates from previous work which relied on significantly more observations, albeit with larger uncertainties. We also note that in addition to measurements from a single observation site, our analysis relied on existing inventories and global models. Further, our analysis of the emissions in five cities suggests that residential volume may serve as a more effective regional proxy for CH_4_ emissions compared to population, reflecting the nuanced composition of urban areas and their specific emission drivers. These findings highlight the importance of tailoring proxies to the unique characteristics of different regions to better understand urban CH_4_ dynamics. For example, Indianapolis’s low emissions could be due to upgrades in metering and regulating stations as well as changes in pipeline materials specific for this urban domain.^61^

Regions with rich measurement networks, such as those analyzed in this study, provide a critical foundation for validating and comparing emission estimation methods. As satellite-based observations and low-cost/medium precision sensor networks become increasingly integral to CH_4_ monitoring, these traditional, high-precision networks will remain essential for benchmarking and ensuring the accuracy of emerging technologies. Here we show that this validation can occur with only a single high-accuracy measurement site if the analysis is conducted appropriately and leverages existing inventories and global CH_4_ models. By doing so, we make the argument that the use of in situ measurements does not require the high costs of associated with establishing and maintaining a dense network of sites.

The approach presented in this study can be feasibly expanded to other urban areas. Several large cities have at least one existing high-quality in situ monitoring site, or the potential for rapid deployment of such instruments. If the observational data are calibrated to internationally recognized standards (e.g., WMO), they can be used in a comparable framework. Furthermore, publicly available meteorological data sets (e.g., ERA5 or HRRR) and gridded emissions inventories (e.g., EPA, EDGAR) make it possible to apply this method in other regions with relatively low implementation costs.

However, expansion depends on the availability of key observational and modeling infrastructure. At a minimum, each target region would require an in situ CH_4_ monitoring location with sufficient temporal coverage. While the technical requirements are modest compared to fully instrumented urban networks, thoughtful site placement, quality assurance, and computational capability are critical to ensure accurate and generalizable emission estimates. With appropriate infrastructure, this framework offers a scalable, cost-effective solution for tracking urban methane emissions internationally.

Integrating complementary observational platforms, such as sector-specific measurements targeting landfills, wastewater treatment plants, and other fugitive methane sources, could significantly enhance emissions attribution and mitigation. The ability to combine a single-site, high-accuracy framework with targeted mobile, remote sensing, and lower-cost/medium precision sensor networks offer a flexible, cost-effective, and scalable path forward for improving urban methane monitoring. By combining these approaches, a comprehensive system can better quantify and mitigate urban CH_4_ emissions and learn more about proxies that correlate to them.

## Supplementary Material



## Data Availability

Atmospheric methane observations used in this study are available at 10.18434/mds2-3012 and https://daac.ornl.gov/cgi-bin/dsviewer.pl?ds_id=1982.

## Notes

The Supporting Information document includes additional details, figures, and tables on observations; atmospheric meteorology and dispersion modeling; transport; methane inventories, modeled enhancements, and wetland emissions; background adjustment analysis; covariance matrices used in the Bayesian Scaling Factor Inversions; inversion filtering, estimation domain evaluation, number of estimates in monthly means, inversion metrics, and estimated scaling factors; and characteristic analysis – data (PDF)
