# Supplementary material for: Scaling Urban Methane Emissions: Utility of Single-Site Measurements in Five Urban Domains
Source: Environ Sci Technol. 2025 Jul 9;59(28):14399–409. doi: 10.1021/acs.est.5c03844 (PMC12288071; doi:10.1021/acs.est.5c03844)
Supplement: Supplementary file 1 [file es5c03844_si_001.pdf]

Supporting Information for
"Scaling Urban Methane Emissions: Utility of Single-Site Measurements in Five Urban Domains"

Kimberly L. Mueller, Anna Karion, Israel Lopez-Coto, Julia Marrs, Vineet Yadav, Genevieve Plant, Joseph Pitt, Zachary R. Barkley, James Whetstone

*Corresponding author email: Kimberly.Mueller@nist.gov

This PDF file includes:

Number of pages: 28
Number of Figures: 23
Number of Tables: 4

Table of Contents:

SI-1: Observations: S2

SI-2: Atmospheric Meteorology and Dispersion Modeling: S3-S5

SI-3: Methane Inventories, Modelled Enhancements, and Wetland Emissions: S5-S9

SI-4: Background Adjustment Analysis: S9-S17

SI-5: Covariance Matrices Used in the Bayesian Scaling Factor Inversions: S17

SI-6: Inversion Filtering, Estimation Domain Evaluation, Number of Estimates in Monthly Means, Inversion Metrics, and Estimated Scaling Factors: S17-S24

SI-7: Characteristic Analysis – Data: S24 -S26

References: S27-S29

SI-1: Observations

The tower-based high-precision in-situ observations of CH_4 dry air mole fraction (units of nmol mol^{-1}, or parts per billion, ppb, henceforward) used in this study (Table-S1) are calibrated to the World Meteorological Organization scale (WMO X2014) for CH_4. We use hourly-averaged observations during local afternoon hours, defined as between 5 hours after local sunrise and before sunset.[1–3] This definition relies on identifying periods with minimal vertical concentration gradients at our tower sites, indicative of a well-mixed planetary boundary layer (PBL). With this definition, the number of hours used in summer is higher than in winter (Fig-S1).

Table-S1: Tower sites whose observations are used in the analysis to estimate emissions either for reported emissions in the manuscript (use in manuscript = "main analysis") or for checking where the use of two towers to estimate emissions in a 50 km radius changes the emissions results (use in manuscript = "analysis check")

Site Code	Full Name	Urban Domain	Latitude	Longitude	AGL (m)	Data Provider	Use in Manuscript
ARL	Arlington	Washington DC	38.892	-77.132	66	Earth Networks	Analysis Check
BUN	Boston University	Boston, MA	42.350	-71.104	29	Boston University	Main Analysis
COP	Copley Tower	Boston, MA	42.347	-71.083	155	Boston University	Analysis Check
MNY	Mineola New York	New York City, NY	40.750	-73.638	76	Earth Networks	Main Analysis
NDC	North DC	Washington DC	38.950	-77.080	91	Earth Networks	Main Analysis
NEB	Northeast DC	Baltimore, MD	39.315	-76.583	67	Earth Networks	Main Analysis
NWB	Northwest DC	Baltimore, MD	39.345	-76.685	55	Earth Networks	Analysis Check
RIC	Richmond	Richmond, VA	37.509	-77.576	95	Earth Networks	Main Analysis

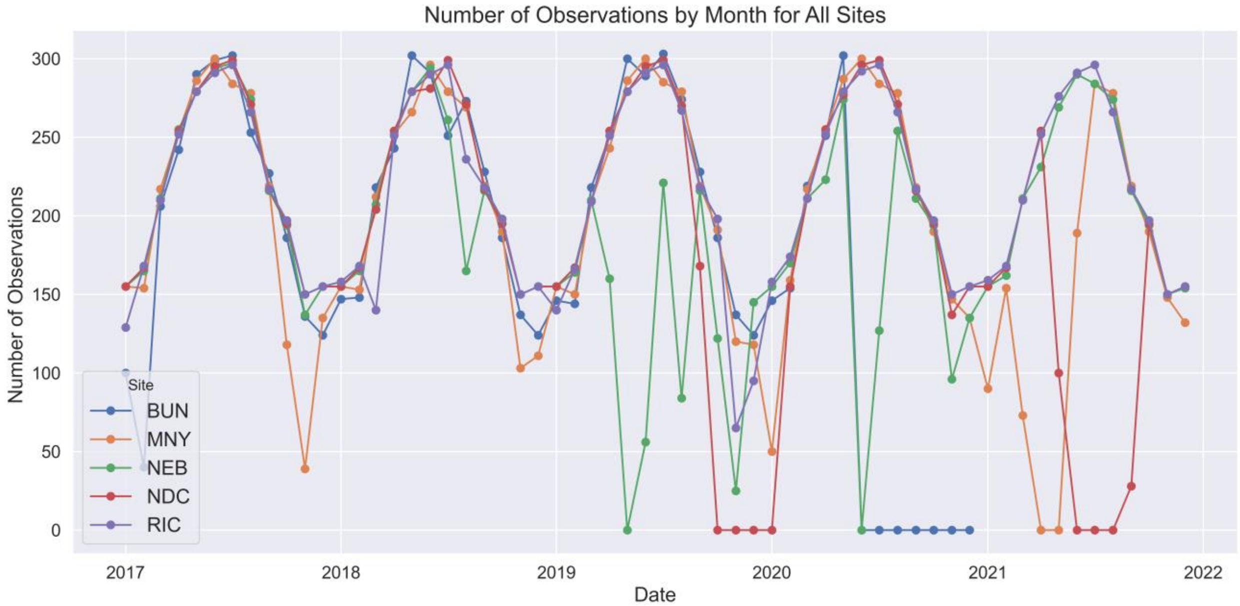

Figure S1: The number of observations from each of the towers in Table-S1, grouped by month, used in the analysis.

SI-2: Atmospheric Meteorology and Dispersion Modeling

The Stochastic Time-Inverted Lagrangian Model (STILT) [4] is used to simulate the backward movement of particles from observation locations in time and space. These simulations create footprints, which show the areas at the surface that contribute to the enhancements observed at each location. Additionally, STILT determines where and when these particles entered the study area before reaching the observation 4-D location. This information is crucial for estimating background, $\mathbf{BG_{gb}}$ (Fig-2).

In this study, we released 960 particles per hour (afternoon hours only) in STILT for each observation point across all towers listed in Table-S1. These particles were released uniformly throughout each hour to represent hourly means and tracked backward for 120 hours. The STILT model was run without a near-field correction (NFC) given that the application of an NFC in STILT had a smaller impact than the difference using WRF and ERA5. Footprints were calculated for a gridded domain with a resolution of 0.1 degrees (d01 in Fig-1). These footprints are used with emission data to estimate the enhancements caused by those emissions. Footprints were calculated for a gridded domain with a resolution of 0.1 degrees (d01 in Fig-1). These footprints are used with emission data to estimate the enhancements caused by those emissions.

To understand the influence of meteorological models on our uncertainty estimates, we used STILT with data from two different models: ERA5 (ECMWF Re-Analysis), and the National Center for Atmospheric Research's Weather Research and Forecast (WRF). ERA5 data is publicly available while the WRF simulations were conducted specifically for this project using the same physics configuration as Karion et al.,[5] for a single domain at 9 km resolution with ERA5 reanalysis for driving forces.[6,7] Additionally, these simulations were nudged towards ERA5 data at hourly intervals. More details about each model are provided in Karion et al.[5].

Note that the annually averaged afternoon footprints from the two meteorological models (coupled to STILT) differ spatially (Fig-S3; Fig-S4). The footprint strengths are also different, where the ERA5 footprints are stronger than those generated from the WRF model (Fig-S5).

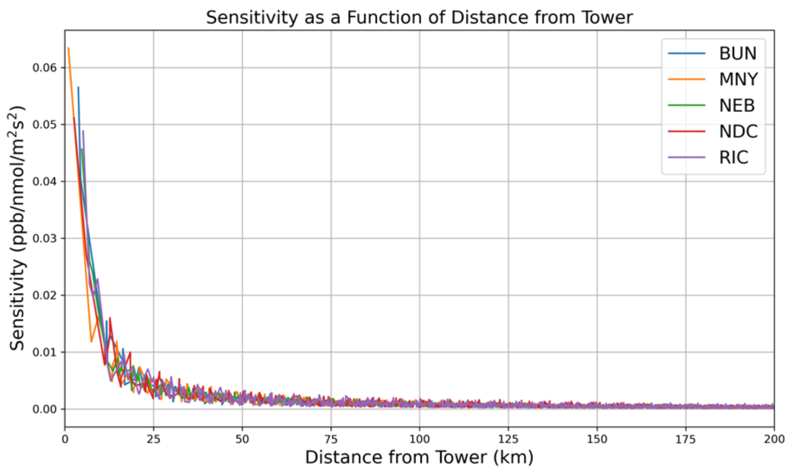

Figure S2: Average afternoon sensitivities (footprints) across the entire estimation period of the study associated with each of the 5 sites, as a function of distance from the site. These sensitivities were generated with WRF-STILT configuration.

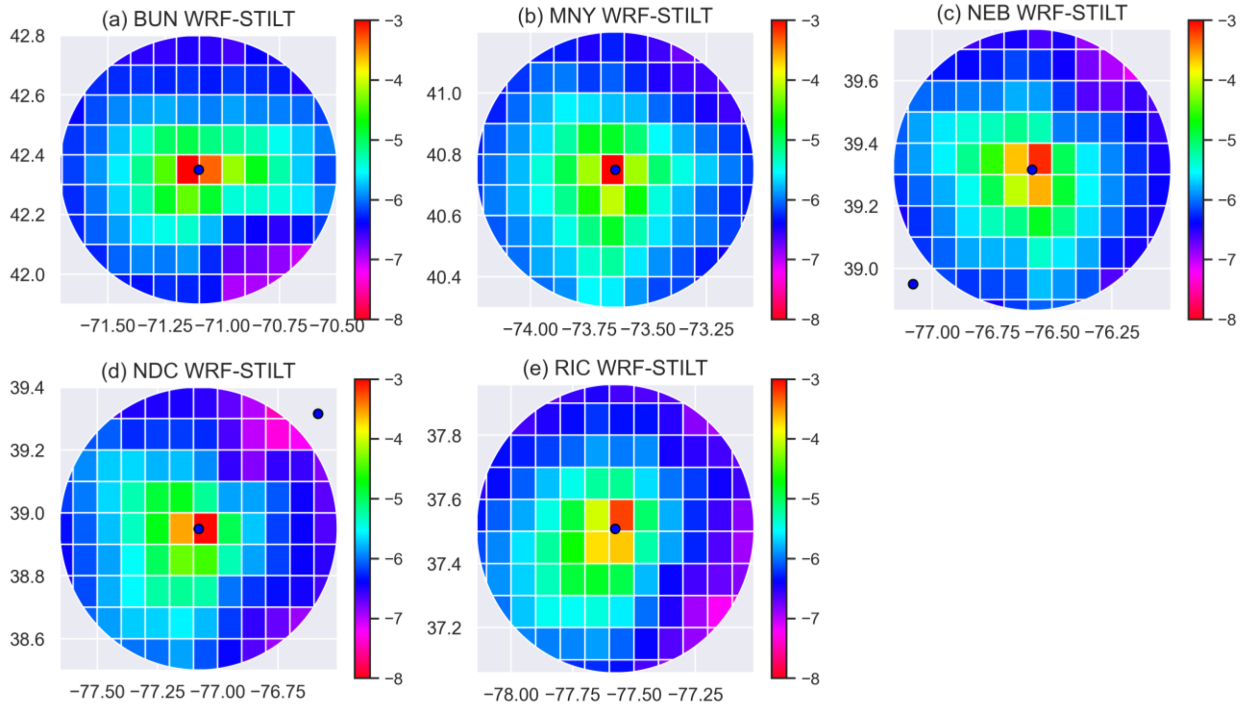

Figure S3: Annual (2018) averaged afternoon WRF-STILT footprints for a domain of 50 km around the observation locations (blue dots) used in the analysis. Units are in log(ppb/nmol/m^{2}s). The grid is 0.1 degrees.

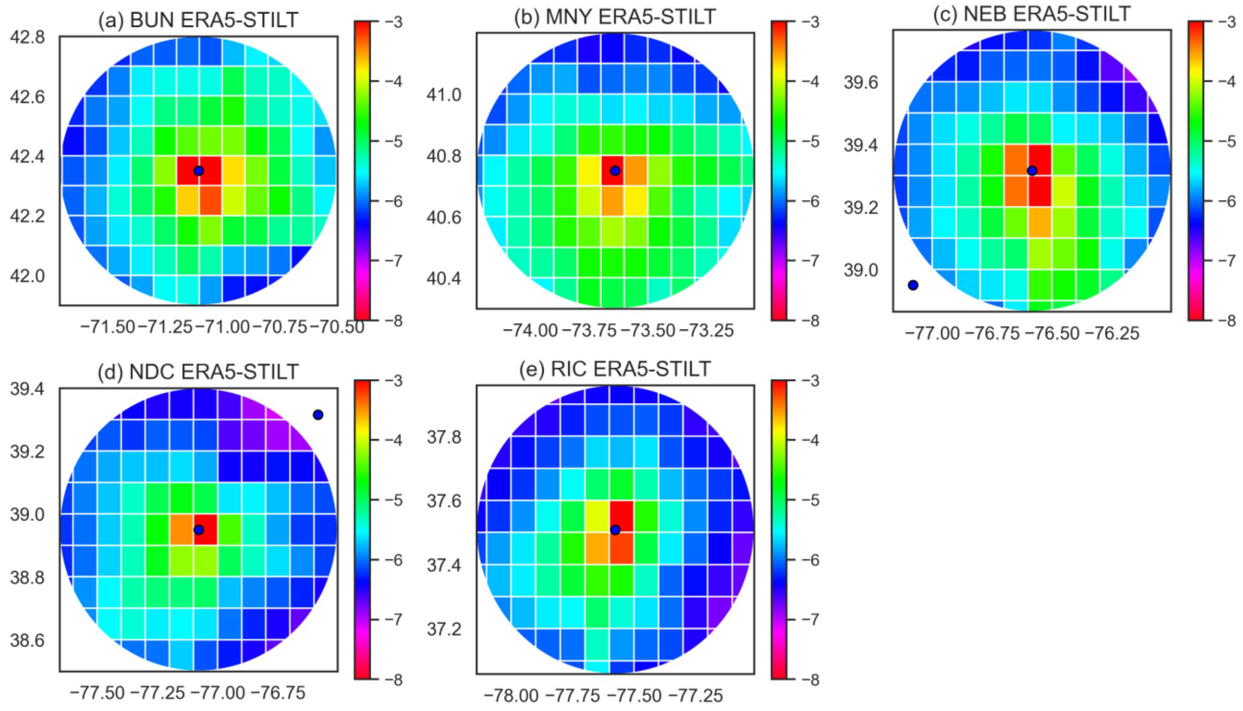

Figure S4: Annual (2018) averaged afternoon ERA5-STILT footprints for a domain of 50 km around the observation locations (blue dots) used in the analysis. Units are in log(ppb/nmol/m^{2}s). The grid is 0.1 degrees.

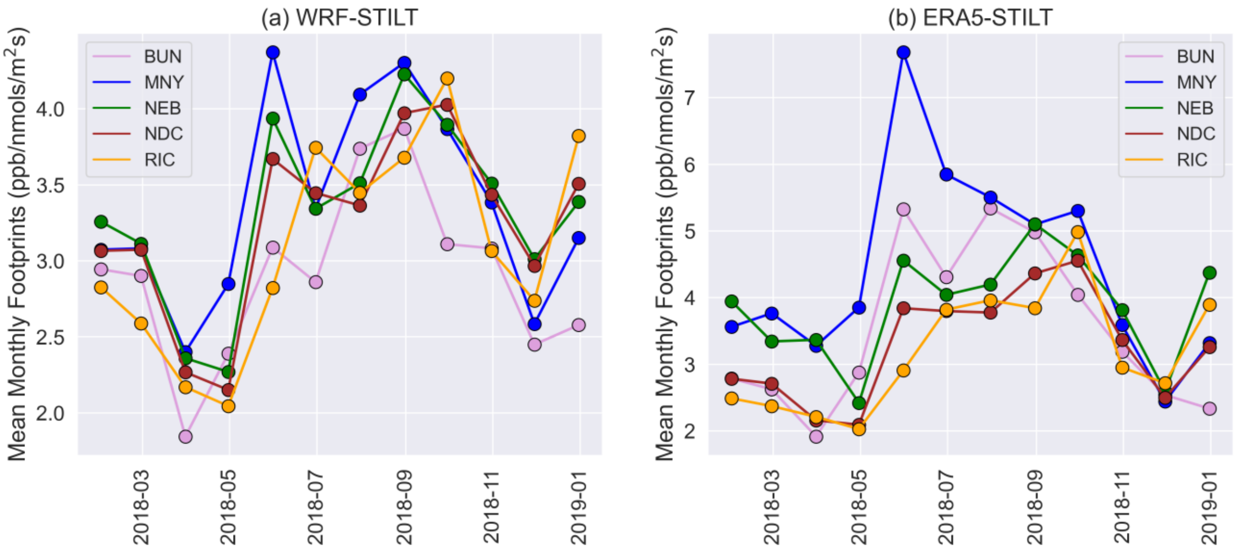

Figure S5: Monthly (2018) averaged afternoon footprints for a 0.1 degree grid cells that intersect a circle with a radius of 50 km around the observation locations used in the analysis. Units are in ppb/nmol/m^{2}s. (a) Footprints generated using the WRF-STILT colored by different towers as indicated in the legend. (b) Footprints generated using the ERA5-STILT.

SI-3: Methane Inventories, Modelled Enhancements, and Wetland Emissions

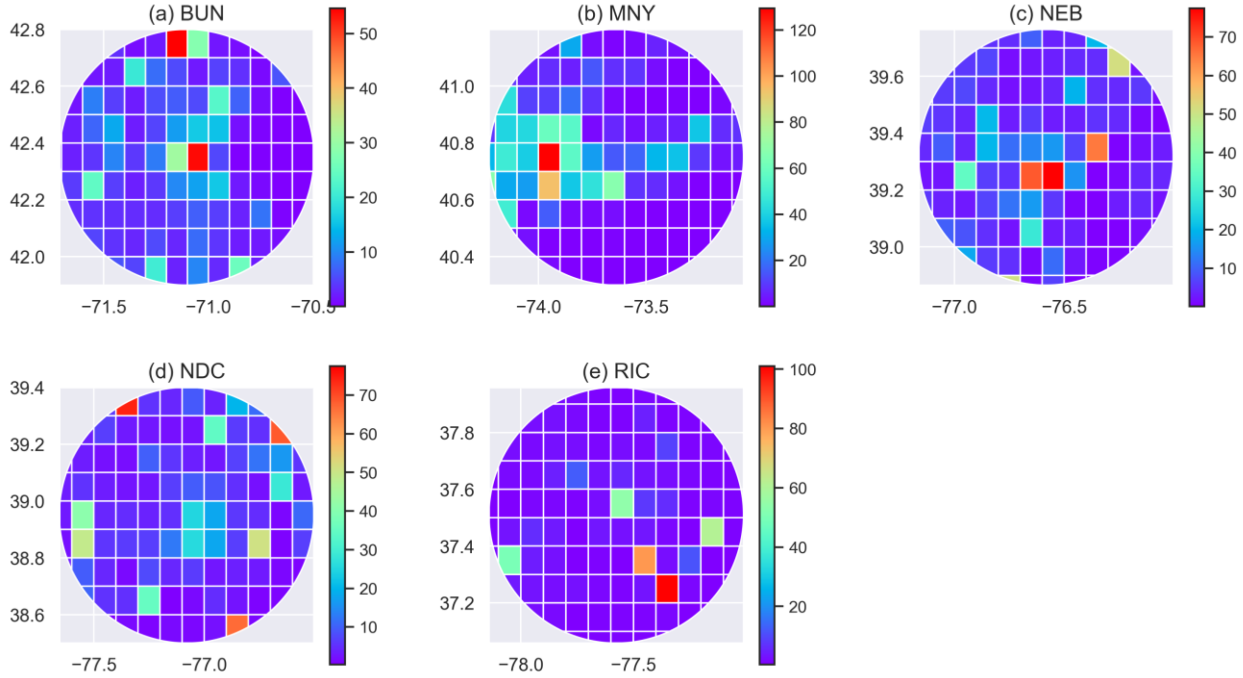

Figure S6: 2018 annual average EPA emissions in 50 km radius from measurement location. Units are nmol/m^{2}s.

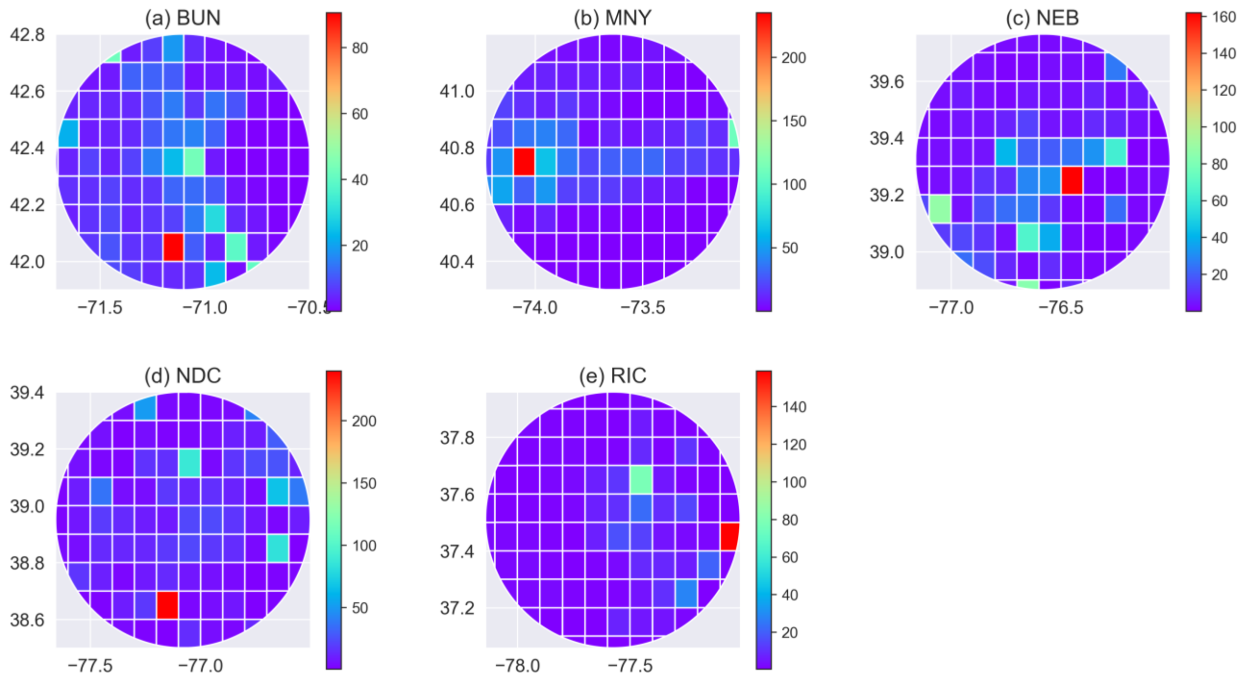

Figure S7: 2018 ED8 emissions in 50 km radius from measurement location. Units are nmol/m^{2}s.

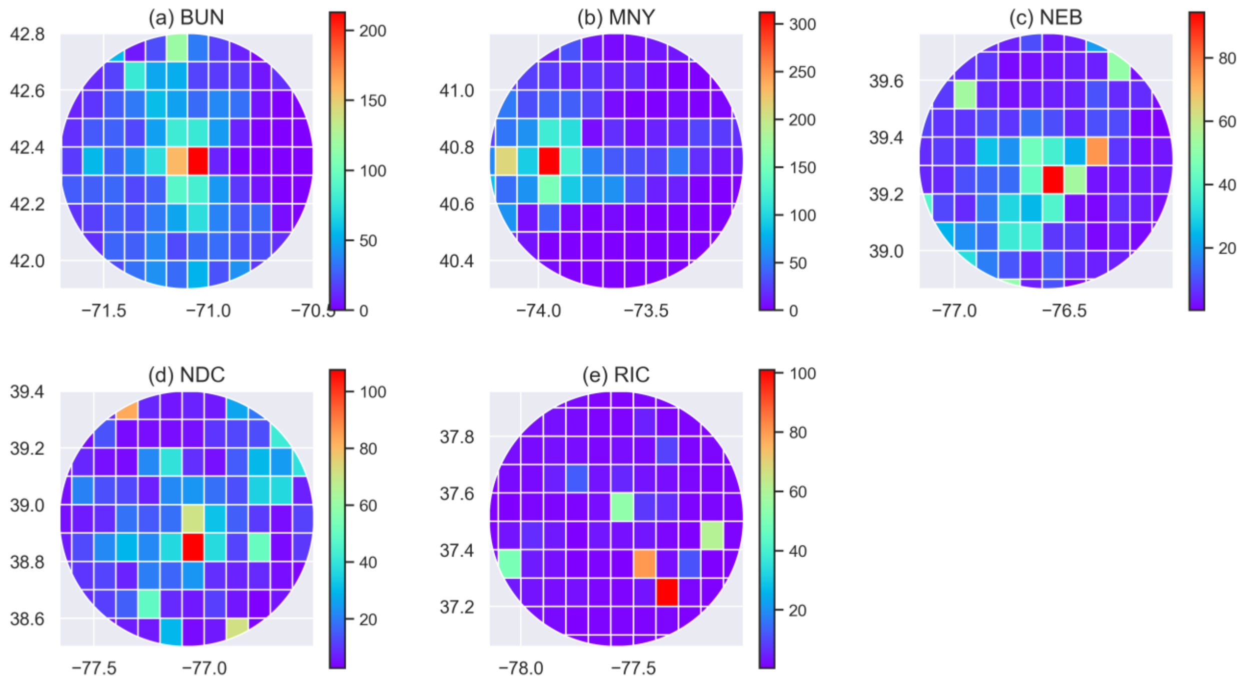

Figure S8: 2018 EmbEPA emissions in 50 km radius from measurement location. These custom emission inventories are from Pitt et al.[8] (MNY), Karion et al., [5] (NEB and NDC), and Sargent et al.[9], (BUN) which are aggregated from their native resolutions to 0.1 degrees. Note EmbED8 are not shown since the distribution of emissions within the 50 km estimation area are largely from the custom inventories, so they look nearly identical to EmbED8. Units are nmol/m^{2}s.

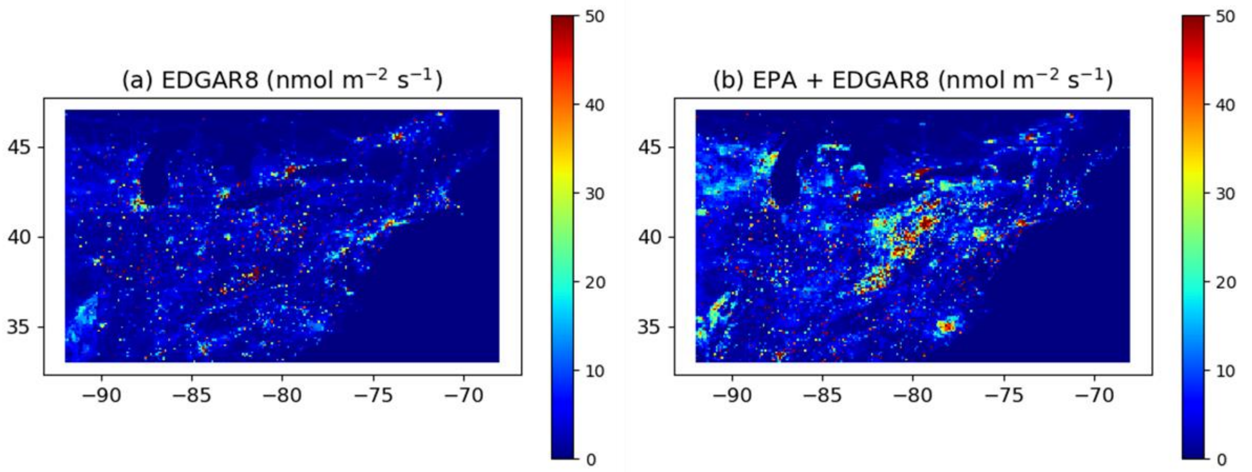

Figure S9: Regional d01 emissions from (a) EDGAR 8 (ED8) and (b) EPA.

Modelled Enhancements:

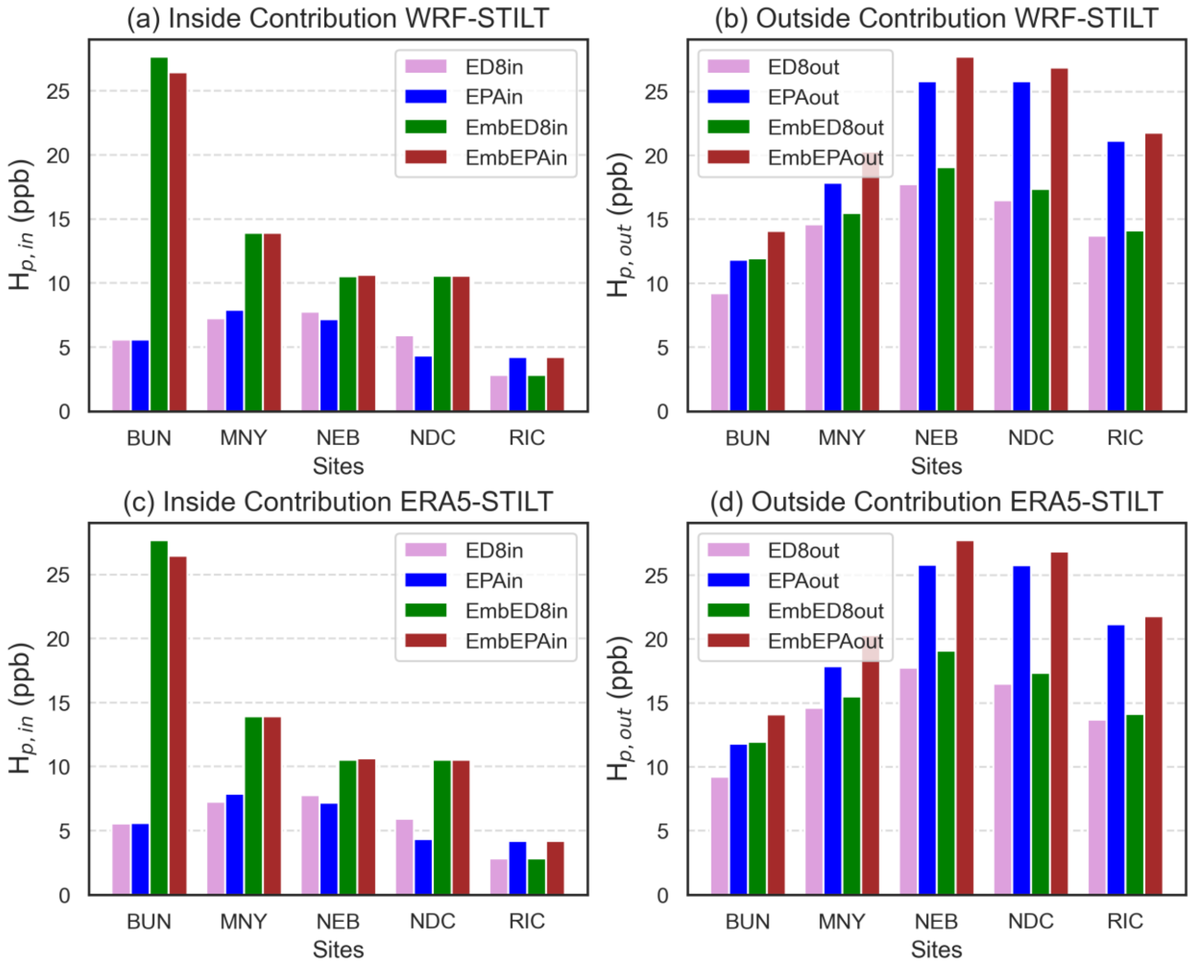

Figure S10: Modelled enhancement - $\mathbf{Hs}_{\boldsymbol{p,in}}$ (left column, a and c) and $\mathbf{Hs}_{\boldsymbol{p,out}}$ **(**right column, b and d) using each emission inventory and the WRF-STILT (top row, a and b) or ERA5-STILT (bottom row, b and c) footprints.

Wetland Emissions within 50 km estimation domain:

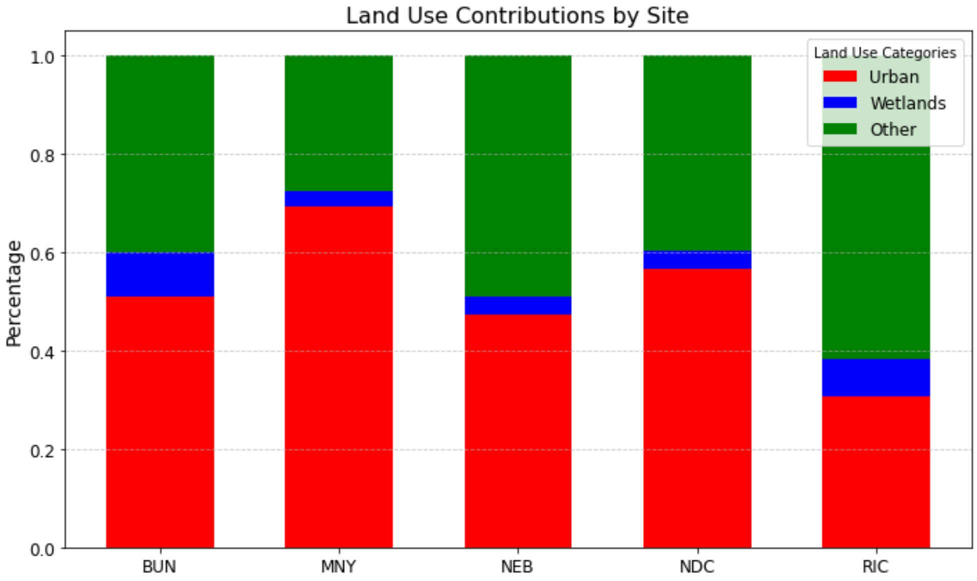

Figure S11: The percentage of convolutions of the National Land Cover Database with the WRF footprints for the 50 km radii. "Urban" is defined as the combination of "Developed, Open Space", "Developed, Low Intensity", "Developed, Medium Intensity", and "Developed High Intensity" classifications.

SI-4: Background Adjustment Analysis

As noted in the main text, the global background contribution to the scaling factor analysis can, potentially, have a large impact on our results. Although the following global background analysis is complex in nature, it does provide a method that can be useful for identifying biases in many other applications and thus, we fully describe the methods herein.

This section is outlined as follows: (1) we compared modelled background mole-fractions from a suite of models to aircraft data to see which background model is the most suitable, (2) because we find that the results of the comparison to aircraft measurement isn't clear cut, we performed a statistical analysis to help identify a background ensemble member that is most suitable and use it to help estimate a bias, (3) after determining a background ensemble member, we used a multi-linear regression and other statistical analysis to help us identify which background towers can help us determine an BG_{adj} (e.g. those that aren't unduly impacted by other sources like emissions or wetlands), (4) to estimate BG_{adj}, we compared to aircraft observations to particle trajectories from these towers and finally, (5) we used a multi-linear regression to confirm whether this offset is consistent the aircraft observations. With this approach, we expect the intercept provides information to help us have confidence BG_{adj} which is a method used by Mead et al.[10]

Motivation:

We performed an analysis to determine whether the variability and the magnitude of typical global backgrounds, like those from the European global Copernicus Atmosphere Monitoring Service (CAMS)[11] which we use within the work presented in the main manuscript are accurate. We used background mole fractions generated using publicly available CAMS 4-D mole fraction fields [v2017; v2018; v2019] with each model year having two products: (1) assimilating remote sensing observations ("r") and (2) assimilating remote sensing observations and in-situ measurements ("r1s"). The CAMS products are global inversion 4-D fields at 4-hourly, 2-degrees by 3-degrees resolution, available at

https://atmosphere.copernicus.eu/greenhouse-gases-supplementary-products last access: 30 December 2024.

While our input data differs somewhat from those in the manuscript (e.g., we use different inventory products and meteorology – aka NAM instead of ERA5, etc.) we believe this approach provides a reasonable initial estimate for adjusted background (BG_{adj}). BG_{adj} is further adjusted using the observations and specific meteorology in the inversion. Note, we also look at CT- CH_4[12](which was released after our initial assessment of BG_{adj}) with a subset of data and found similar results that are not shown here.

To assess which CAMS product best explains the true variability of the observations and determine if there is a potential bias in the $\mathbf{BG_{gb}}$, we begin by exploring an assortment of CH_4 emission products in d01 (Table-S3) and observed CH_4 mole-fractions using the towers in Table-S1.

Table-S2: Emission products to estimate a prior BG_{adj}. Each uses different assumptions for spatially downscaling CH_4 emissions. For example, ED4.2 uses population to downscale national CH_4 emissions. The star next to EPA indicates that it was embedded into ED6 to include Canadian emissions. Note, these inventories are not the same products used in the main scaling analysis because many in the manuscript were available after this analysis.

Emission Product	Representative Year	Source	Temporal Resolution
ED4.2	2018	Janssens-Maenhout et al.[13]	Annual
ED4.3.2	2010	Janssens-Maenhout et al. (2019)[14]	Monthly
ED5	2015	Ferrario et al.[15]	Monthly
ED6	2016	Crippa et al.[16]	Monthly
EPA*	2012	Maasakkers et al. (2016)	Monthly
Mean	mix	mix	Monthly

We know that, if the meteorological and global CH_4 models, as well as the CH_4 emission products are unbiased and precise, the enhancements from all sources in d01 is:

$$\mathbf{Enh_{d01}} = \boldsymbol{y_{twr}} - \mathbf{BG}_{\boldsymbol{gb}} \qquad \text{Eq. S1,}$$

Which can be approximated as:

$$\mathbf{Enh_{d01}} = \mathbf{Hs} \qquad \text{Eq. S2}$$

However, as mentioned earlier, there are many uncertainties and potential biases associated with $\mathbf{BG_{gb}}$, with the transport and dispersion model footprint, or Jacobian (**H**), and with the emissions $\mathbf{s} = \mathbf{s}_{\boldsymbol{p,out}} + \mathbf{s}_{\boldsymbol{p,in}}$. This is illustrated by plotting the regional enhancements ($\mathbf{Enh}_{d01}$) at a tower location (Fig-S12). We generate $\mathbf{Enh}_{d01}$ subtracting $\mathbf{BG_{gb}}$ (i.e., CAMS products) from the observed CH_4 mole fractions and plotting them against convolutions (**Hs**). In Fig-S12, we compare the spread in the convolutions (**Hs**) to the spread in $\mathbf{Enh}_{d01}$. We can also see how the magnitudes align. In theory, $\mathbf{Enh}_{d01}$ should be of similar magnitude as one of the convolution members which would lend confidence in one of the modelled emissions and the simulated atmospheric transport and dispersion.

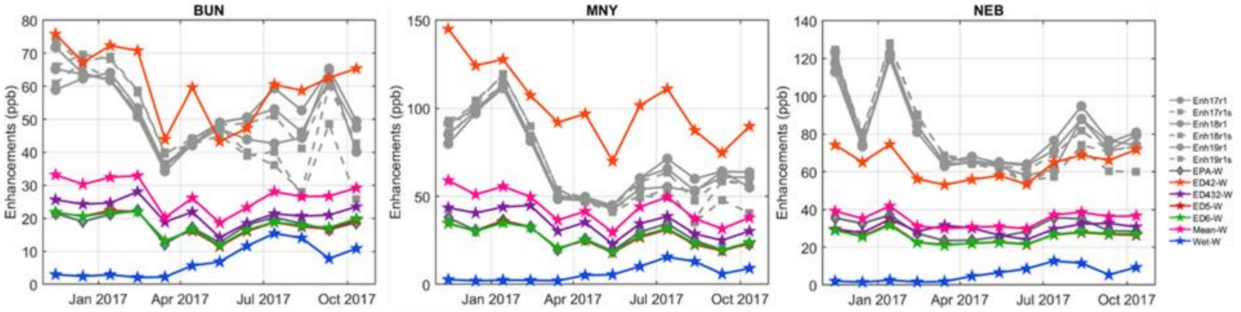

Figure S12: Enh$_{d01}$ using six different CAMS ensemble members (grey) as derived Eq-S1, along with modelled enhancements (**Hs**) (in colors) using **H** from WRF-STILT and different emission products (**s**) as indicated by the legend on the right side of the figure (and listed in Table-S2) at three different urban towers (BUN, MNY, and NEB as indicated by each panel's title).

The large magnitude of all the **Enh**$_{d01}$ compared to the modelled enhancements (**Hs**) as well as the spread of the **Enh**$_{d01}$ members, especially in late Spring to mid-Fall gives us pause for consideration. The most probable reasons why the **Enh**$_{d01}$ are much larger than all but one **Hs** member is that (1) the $\mathbf{BG_{gb}}$ values are all too low, (2) the emission products are too low, (3) there is a significant bias in the atmospheric transport and dispersion model or (4) some combination of the three. Even though we have limited knowledge about the accuracy of regional emission products, our understanding of the ability of the CAMS global model to provide an accurate $\mathbf{BG_{gb}}$ is even more lacking. The spread of the CAMS ensemble in late-Spring to mid-Fall is an indication that the uncertainty in the CAMS background, during this time, is large. Thus, we investigate other data sources, specifically associated with $\mathbf{BG_{gb}}$, and methods that may shed light on the accuracy and precision of the CAMS model at the edge of our regional domain and whether we can select the CAMS ensemble member that best explains the variability in the in-situ observations.

Step 1: Background Assessment using Aircraft Observations

The National Oceanic and Atmospheric Administration's Global Monitoring Laboratory (NOAA/GML) provides routine aircraft observations[17] of CH_4 at different vertical heights within the atmosphere at six locations near the edge of our regional domain (Fig-S11). For each observation location, we interpolate the 4D CAMS fields in space and time to the observation location and time and compare the observed profiles with the CAMS globally modelled CH_4. Ideally, the CAMS data should match the observed CH_4. If not, we would hope that the difference be unbiased and randomly vary around the observed CH_4 mole factions.

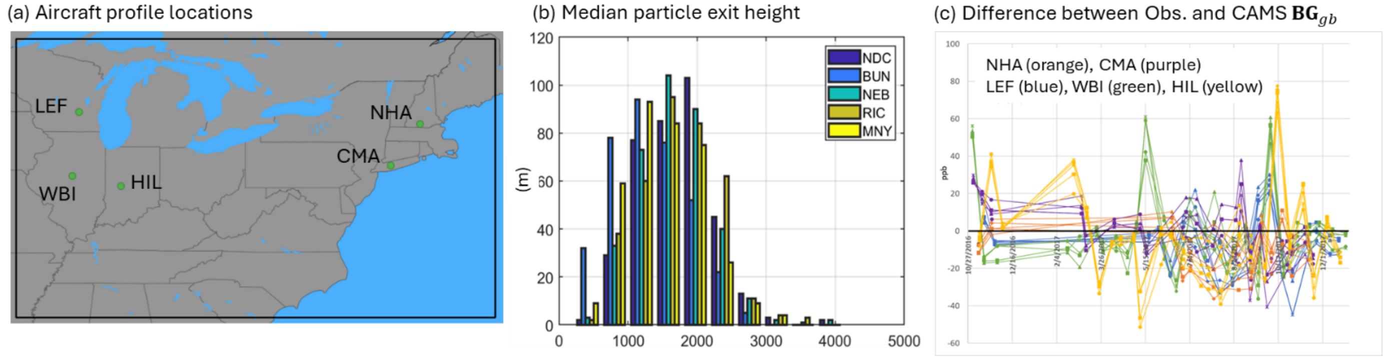

Figure S13: (a) Map of our d01 and aircraft sampling locations (green circles). Ideally, NOAA flies around each sampling location every two weeks, but large data gaps exist. (b) mean particle exit heights at the heights where they leave d01. (c) aircraft observation minus the CAMS backgrounds across 2017.

However, when we look at the difference between the aircraft observations and the CAMS products at each of the aircraft observing locations (Fig-S13a) indicates that all CAMS products are generally too high (Fig-S13c) in the mid-troposphere, aka 1,500 m, which is the average exit height when the particles, as released from our urban tower locations, leave d01. When looking through all the comparisons between the flight profiles and the CAMS products, it is difficult to ascertain which CAMS product is closest to the observations (due to gaps, different sampling locations, etc.). However, when taking the mean at 1500 m, CAMS17r1s and CAMS17r1 appears to be slightly better than the others but within the variability of the other means (results not shown). But these results are far from clear given the variability and thus, additional analysis is warranted to assess which CAMS product best reproduces the variability in the observations and to investigate whether an overall offset can be estimated to correct this bias.

Step 2: Identifying BG_{gb} Ensemble

Mathematical Theory:

In theory, a regression expresses the dependent variable (e.g., in this case $\boldsymbol{y_{twr}}$) as the sum of a deterministic component and a stochastic term. The deterministic component represents the part of the observed CH_4 mole fractions that can be explained using a linear combination of set of covariates. The deterministic component takes the form of a model of the trend (i.e., $\mathbf{X\beta}$) where the $\mathbf{X}$ matrix contains covariates whose specific elements will be discussed later in the text. These vectors are scaled by unknown coefficients ($\boldsymbol{\beta}$). Any assumed structure between $\hat{\boldsymbol{y}}_{twr}$ and $\boldsymbol{y_{twr}}$,($\boldsymbol{y_{twr}}$ - $\hat{\boldsymbol{y}}_{twr}$), can be captured in a covariance matrix $\mathbf{P}$. The regression is generally used to obtain the best estimate of the unknown drift coefficients, $\hat{\boldsymbol{\beta}}$, and their corresponding variance, $\boldsymbol{\sigma^2_{\hat{\beta}}}$.

$$\boldsymbol{y_{twr}} = \mathbf{X\beta} + \mathcal{E} \qquad \text{Eq. S3,}$$

$$\boldsymbol{y_{twr}} = \beta_0 + \beta_1\mathbf{X_1} + \beta_2\mathbf{X_2} + \beta_3\mathbf{X_3} + \beta_4\mathbf{X_4} + \mathcal{E} \qquad \text{Eq. S4,}$$

Bayes Information Criteria (BIC)[18] and adjusted R^2 provide metrics for selecting the best sets of covariates by ranking how well each column of $\mathbf{X}$ explains the variability in $\boldsymbol{y_{twr}}$. However, we cannot rely on the BIC metric alone since many of the columns in $\mathbf{X}$ are correlated. This results in likelihoods that are not significantly different between models. Nonetheless, the BIC metric helps us narrow the number of candidate models so that we do not need to examine every regression subset. To complement the BIC analysis, we use adjusted R^2 to us determine which group of covariates help explain $\boldsymbol{y_{twr}}$.

To calculate BIC, we first estimate the residual sum of the squares (RSS) so that the BIC of a model, $\mathbf{X}$, with k columns (where k = 1 … 4) is:

$$RSS = \boldsymbol{y_{twr}}(\mathbf{P}^{-1} - \mathbf{P}^{-1}\mathbf{X}(\mathbf{X}^T\mathbf{P}^{-1}\mathbf{X})^{-1}\mathbf{X}^T\mathbf{P}^{-1})\boldsymbol{y_{twr}} \qquad \text{Eq. S5}$$

$$BIC = \ln|\mathbf{P}| + RSS + k\ln(n) \qquad \text{Eq. S6}$$

where n is the number of observations, $\boldsymbol{y_{twr}}$.

The estimated coefficients, $\hat{\boldsymbol{\beta}}$, for each column in $\mathbf{X}$ and their associated uncertainty covariance ($\mathbf{V_{\hat{\beta}}}$) are calculated using:

$$\hat{\boldsymbol{\beta}} = (\mathbf{X}^T\mathbf{P}^{-1}\mathbf{X})^{-1}\mathbf{X}^T\mathbf{Q}^{-1}\boldsymbol{y_{twr}} \qquad \text{Eq. S7}$$

$$\boldsymbol{V_{\hat{\beta}}} = (\mathbf{X}^T\mathbf{P}^{-1}\mathbf{X})^{-1} \qquad \text{Eq. S8}$$

Analysis:

A general workflow of our analysis is to identify the CAMS member to use as $\mathbf{BG_{gb}}$, and to investigate whether an offset can be quantified. First, we averaged daily afternoon observations from towers in the regional domain (28 towers). For afternoon hours, we used a sunrise and sunset criteria (see section S1) to focus on periods when the atmosphere is well-mixed, i.e., when we assume we can best trust modelled meteorology and dispersion. We used daily averaged observations as we expect the variability of the hourly observations to be large enough to obscure the ability of the CAMS model to explain the overall variability in $\boldsymbol{y_{twr}}$. Later, we used Cook's statistics to remove any outliers in $\boldsymbol{y_{twr}}$ that may impact our assessment.

After averaging all afternoon observations to have a daily average, we identified the CAMS ensemble to use as $\mathbf{BG_{gb}}$ per method described henceforward in this section.

We performed a variable selection where we estimate BIC and R^2 for different formulations of $\mathbf{X}$. That is, we used every possible combination of different ensembles in $\mathbf{X}$ but fix each column to represent a specific component that explains the variability in $\boldsymbol{y_{twr}}$. Note, the Jacobians $\mathbf{H}$ are constructed using WRF-STILT or NAM-STILT (NAM is the North American Mesoscale Model, https://registry.opendata.aws/noaa-nam). $\mathbf{X}$ is made up of five columns which represent: (1) a global background ensemble, (2) a meteorology-dispersion unit flux ensemble (aka a unit emission in all grid cells multiplied by each $\mathbf{H}$), (3) an emission inventory (Table-S2) ensemble multiplied by each Jacobian ($\mathbf{Hs}$), (4) wetland fluxes (as described in the manuscript) multiplied by each Jacobian ($\mathbf{Hs}_{wet}$) and (5) an intercept. Thus, $\mathbf{X}$ is always a $(nx5)$ matrix (referred as the full model), where n is the number of daily averaged CH_4 mole fractions observed at one of the 28 regional towers. For the residuals ($\mathbf{P}$), we used the sub-hourly variance of the observations $\boldsymbol{y_{twr}}$ along the diagonal. We estimated a scaling factor for the diagonal of ($\mathbf{P}$) using Restricted Maximum Likelihood[19] to translate the sub-hourly variation proxy into a more realistic representation of residual error.

The BIC and R^2 analysis indicate that either CAMSv17r1s or CAMSv17r1 best explain the variability in the observed CH_4 mole-fractions at almost all 28 towers. That is, these ensemble members were selected 86% of time using the model selection with the full model. As a check, we used a regression model using only an intercept and with every possible combination of the CAMS ensembles, or six regressions, and either CAMSv17r1s or CAMSvr1 is selected 96% of time. Note that, as expected, within the full module and depending on a tower location in d01, the CAMS backgrounds largely are not the dominant variable, and they do not generally explain as much of the variability of $\boldsymbol{y_{twr}}$ as the emission convolutions ($\mathbf{Hs}$) or the unit flux convolutions. In most instances, the convolved EPA emissions are the dominant variable. Even though the selection of CAMSv17r1s and CAMSv17r1 is not always statistically significant for the 28 model selection results, it is consistent and not influenced by outlier events or large observational data gaps that exist at certain towers. This analysis allows us to confidently use either CAMSv17r1s or CAMSv17r1 to assess the bias associated with $\mathbf{BG_{gb}}$. From henceforward, we use CAMSv17r1s.

Step 3: Selecting Background Sites to Determine $\mathbf{BG}_{adj}$

Even though CAMS17r1s may best the model for $\mathbf{BG_{gb}}$ that explains the variability in $\boldsymbol{y_{twr}}$, it still is biased high as implied by the aircraft vertical profile plots. We explored whether we could estimate an adjustment ($\mathbf{BG}_{adj}$) so that $\mathbf{BG_{gb}}$ is more in line with both aircraft and in-situ site data. A substantial positive bias will cause us to underestimate enhancements and thus, infer lower emissions. We investigated an offset by first identifying regional towers (excluding those in urban domains) where $\mathbf{BG_{gb}}$ has the most importance in the full model $\mathbf{X}$ $(nx5)$ in their ability to explain the observations. In this case, the full model includes the selected variables for each tower as identified in our previous analysis. Note that importance is a unitless

number that ranges from 0 to 1 as explained in Yadav et al.,[20]. We looked for instances when $\mathbf{BG_{gb}}$ (aka, CAMS17r1s) has a large importance (Fig-S14 and Fig-S15, DVA, MNC, SO9, LEF, DNC, UNY, and SKY) compared to $\mathbf{Hs}$ ($\mathbf{s}$ is the EPA emissions) and $\mathbf{Hs}_{wet}$. For all these locations, the importance metric of the $\mathbf{BG_{gb}}$ component is 0.35 or more. The importance of a covariate within a regression model tells us how much information is contained within a covariate within the deterministic component of the regression ($\mathbf{X\beta}$) based on how orthogonal the variable's information is from the others.

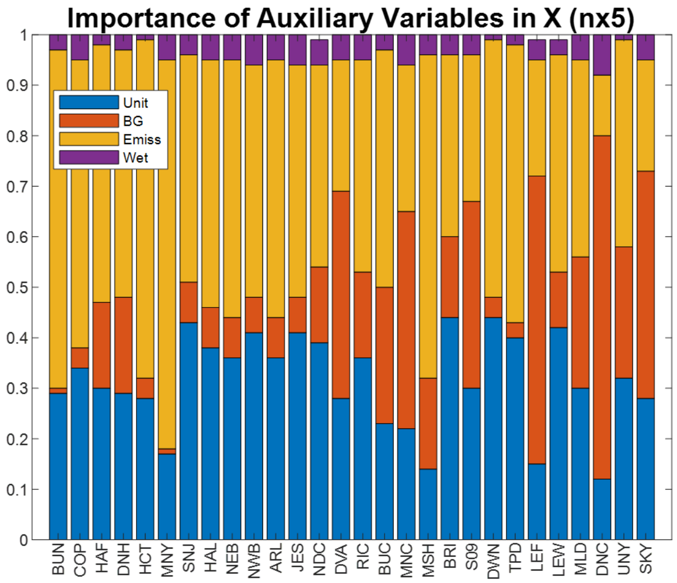

Figure S14: The importance of each column in **X** (excluding intercept). Unit (blue) is the convolved unit fluxes component, orange is the $\mathbf{BG_{gb}}$ component, Emiss. (yellow) is the (**Hs**) component (EPA emissions), and Wet (purple) is the $\mathbf{Hs}_{wet}$ component.

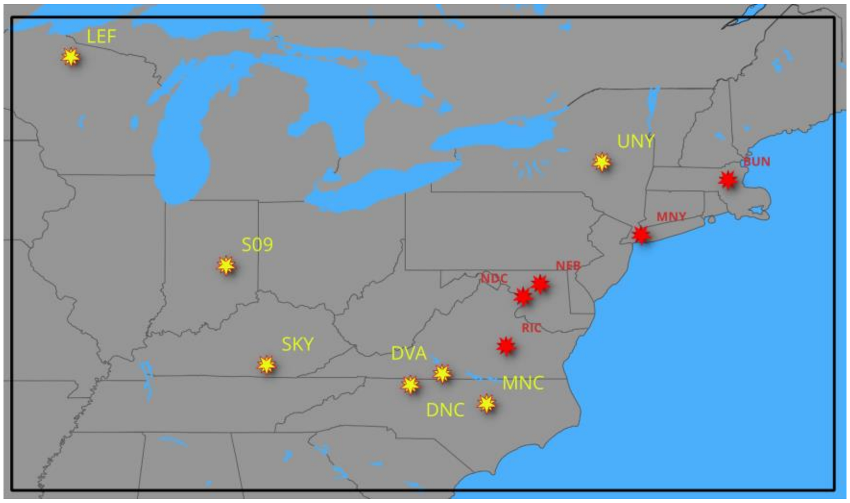

Figure S15: Location of regional measurement tower sites (yellow stars; three letter code) used to assess background adjustment. The red stars show the measurement locations used to estimate methane emissions as shown in the main manuscript. Note LEF in this case is a NOAA/GML tower site ([17]), not the same LEF data shown in the aircraft comparison in Figure S11. Also, note that S09 is a tower owned and operated by Pennsylvania State University[21].

The importance metric cannot be the only measure from which we select a regional background tower site to evaluate a BG_{adj} as any of the covariates within the full model are colinear and thus, do not independently contribute to $\hat{\boldsymbol{y}}_{\boldsymbol{twr}}$. That is, it may be a combination of covariates that statistically significantly explain the variability in $\boldsymbol{y}_{\boldsymbol{twr}}$, including the intercept with other covariates. One means to assess collinearity within a regression model is to examine the correlation between the uncertainties of the correlation ($\boldsymbol{Cov}_{\hat{\boldsymbol{\beta}}}$) between the estimated regression coefficients ($\hat{\boldsymbol{\beta}}$) within the regression model. The covariances can be converted into correlation coefficients using the uncertainties associated with $\hat{\boldsymbol{\beta}}$, aka $\mathbf{RhoV}_{\hat{\boldsymbol{\beta}}}$ for easier interpretation. We refer to these correlation coefficients as $\mathbf{RhoV}_{\hat{\boldsymbol{\beta}}}$ (a $5x5$ symmetrical matrix with a diagonal of 1) – also defined in Eq-S8. If a covariate is highly correlated/anti-correlated with another covariate within the regression model, their corresponding $\mathbf{RhoV}_{\hat{\boldsymbol{\beta}}}$ will be close to positive/negative one.

To estimate $\mathbf{RhoV}_{\hat{\boldsymbol{\beta}}}$ we use the convolved EPA emission product, CAMS17r1s, and the $\mathbf{Hs}_{wet}$ emissions as covariates within the regression model. Instead of running each tower separately, we use all the chosen sites (DVA, MNC, SO9, LEF, DNC, UNY, and SKY) together within the model. We ran the regression estimating a scaling factor for **P** of diagonal of ones to account for the different uncertainties associated for each tower within the regression. We did not use sub-hourly variance of the observations $\boldsymbol{y}_{\boldsymbol{twr}}$ along the diagonal, as before, as this configuration did not improve statistics or impact the uncertainty associated with $\hat{\boldsymbol{\beta}}$. We also setup the regression to estimate an individual intercept number for each tower, i.e.,

$$\mathbf{X} = \begin{pmatrix} 1_{twr\_1} & 0 & 0 & 0 & 0 & \mathbf{CAMSr1s}_{twr\_1} & \mathbf{Hs}_{EPA\_twr\_1} & \mathbf{Hs}_{Wet\_twr\_1} \\ \vdots & \vdots & \vdots & \vdots & \vdots & \vdots & \vdots & \vdots \\ 0 & 1_{twr\_2} & 0 & 0 & 0 & \mathbf{CAMSr1s}_{twr2} & \mathbf{Hs}_{EPA\_twr\_2} & \mathbf{Hs}_{Wet\_twr\_2} \\ \vdots & \vdots & \vdots & \vdots & \vdots & \vdots & \vdots & \vdots \\ 0 & 0 & 1_{twr\_n} & 0 & 0 & \mathbf{CAMSr1s}_{twr\_n} & \mathbf{Hs}_{EPA\_twr\_n} & \mathbf{Hs}_{Wet\_twr\_n} \end{pmatrix} \qquad \text{Eq. S-8}$$

So that,

$$\hat{\boldsymbol{y}}_{\boldsymbol{twr}} = \hat{\beta}_{0\_twr\_1} + \hat{\beta}_{0\_twr\_2} + \cdots + \hat{\beta}_{0\_twr\_n} + \hat{\beta}_1\mathbf{CAMS17r1s} + \hat{\beta}_2\mathbf{Hs_{EPA}} + \hat{\beta}_3\mathbf{Hs_{Wet}} \qquad \text{Eq. S-9}$$

where $\hat{\boldsymbol{y}}_{\boldsymbol{twr}}$ are the estimated CH4 mole fractions from the regression model.

After looking at residuals adjusted R^2, and the assessment of the stability regression model, we determine that DVA, SO9, DNC, UNY, and SKY were the most appropriate background towers to use in our analysis.

Step 4: Using Aircraft Observations to Estimate ($\mathbf{BG}_{adj}$)

With our five regional tower sites selected, we return to the aircraft data. To investigate an offset, we look at the average exit location, including time and space (x, y, and z), of our hourly particles released as part of STILT-NAM or STILT-WRF simulations at each of the five selected regional tower sites. Even though there are several NOAA/Global Modeling Laboratory (GML) aircraft sites across the edges of our d01 domain (Fig-S13), we only examine those where the exit particles leave in the vicinity of the aircraft sampling locations (Fig-S13) at 1500 m.

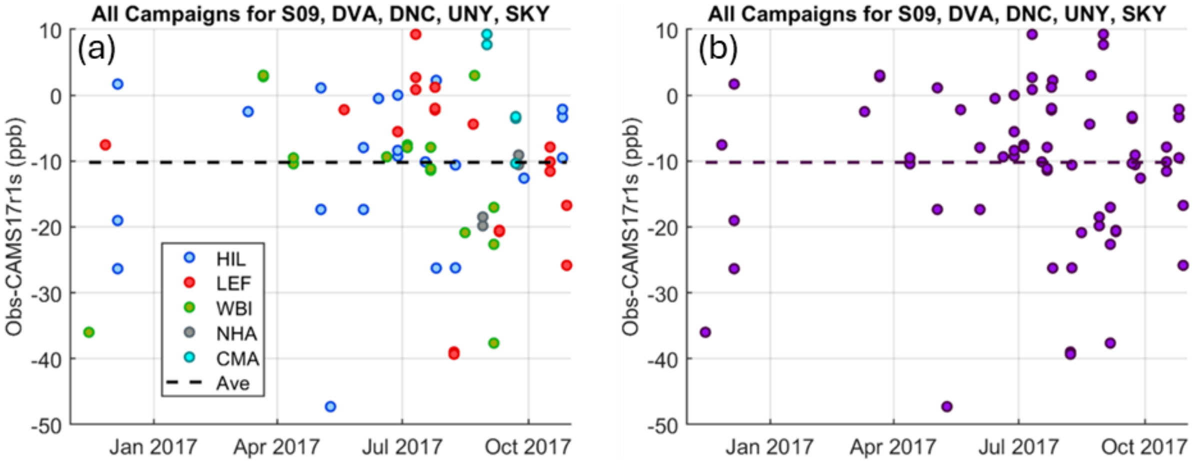

Figure S16: The difference between CH_4 observations observed at 1500 m from the NOAA aircraft (sites indicated in the legend) and CAMS17r1s during times and locations of particles exiting the domain from receptors at the five regional towers. (a) shows the difference for all the sites (color coded for each site) while (b) is the same but just color-coded purple for further clarity. The dashed line shows the mean offset of -10.2 ppb.

This analysis indicates that CAMS171rs may be biased high by ~10.2 ppb (dashed line in Fig S13) at the annual scale. Most of the aircraft data comparison uses sites in the North and Northwest although we assume this offset is consistent across the entire d01 regional domain.

(5) Use a Multi-linear Regression to Confirm $\mathbf{BG}_{adj}$

Because -10.2 ppb is a large offset relative to enhancements, we return to our regression analysis to further support this result. Such a large offset in $\mathbf{BG_{gb}}$ would suggest that the actual emissions are much larger than the convolved emissions.

Using background towers, we anticipate that any offset associated with $\mathbf{BG_{gb}}$ would largely be captured by the intercept term within the regression model, aka:

$$\hat{\boldsymbol{y}}_{twr} = \hat{\beta}_0 + \hat{\beta}_1\mathbf{X_1} + \hat{\beta}_2\mathbf{X_2} + \hat{\beta}_2\mathbf{X_3} + \hat{\beta}_2\mathbf{X_4} \qquad \text{Eq. S-10}$$

where $\hat{\beta}_0$ is the estimated intercept term in units of ppb. $\mathbf{X_1}$ are the unit fluxes, $\mathbf{X_2}$ is the selected CAMS member (CAMS17r1s), $\mathbf{X_3}$ is the selected EPA convolved emission product, $\mathbf{X_4}$ is the selected convolved wetland product, and all the associated estimated regression coefficients are $\widehat{\boldsymbol{\beta}}_{1:4}$. We note that any offset associated with any one of the covariates besides the selected CAMS member would also be captured in the intercept term.

Encouragingly, the intercept terms for all the regional tower sites are statistically similar at 1-σ which supports our assumption of an offset being relatively consistent across the regional domain. Second, the estimated $\widehat{\boldsymbol{\beta}}$ associated with CAMS17r1s is less than one suggesting that the variability in the CAMS background may be too large and its overall magnitude too high. Third, the $\mathbf{RhoV}_{\hat{\beta}}^{2}$ entries associated with the intercept and the CAMS17r1s background are negative one indicating that any offset would be fully represented in the intercept term. Finally, the $\mathbf{RhoV}_{\hat{\beta}}^{2}$ of CAMS17r1s with any other covariate is negligible suggesting that CAMS17r1s and the convolved EPA emissions operate more-or-less independently within the regression model.

To check to see if the -10.2 ppb offset as estimated using the aircraft observations is consistent with the regression, we first use the $\mathbf{RhoV}^{2}_{\hat{\beta}}$ with the mean of intercepts (424 ppb) to create standardized weights to help proportion the mean intercept into two parts, one associated with CAMS17r1s, and the other with the convolved EPA emissions. Note that the intercept is the mean bias when all covariates are zero. We add this proportion of the mean intercept to $\hat{\beta}_1\mathbf{CAMS17r1s}$ to estimate an adjusted CAMS17r1s. Taking the difference of the medians of both CAMS17r1s and its adjusted values, yields an annual difference of ~-13 ppb which is higher but similar in magnitude to the -10.2 ppb offset estimated earlier based on the aircraft analysis. There are other biases captured by these intercept terms, e.g., transport and dispersion error, and thus it is not surprising that this number is higher. Plotting the values of the original CAMS17r1s and the associated adjusted values hints that an offset is dependent on the magnitude of the background values and thus may be seasonally varying. However, an analysis including much more data over many years would be needed to make such a claim. Indeed, the analysis of $\mathbf{RhoV}^{2}_{\hat{\beta}}$ as shown herein provides more of a check on our aircraft analysis rather than a conclusive means to estimate an offset for CAMS17r1s. However, using this method, we find that that the adjusted convolved EPA values are 2.4x their original values which is in line with previous reported values providing us some additional confidence in these results.

In the manuscript, we use -10.2 as our prior adjustment offset $\mathbf{BG_{gb}}$.

SI-5: Covariance Matrices Used in the Bayesian Scaling Factor Inversions

Here, $\boldsymbol{y_{twr}}$ is a vector of the afternoon hourly observations and the $\mathbf{R}$ is the model-measurement mismatch error covariance matrix which characterizes errors associated with measurement, transport, background, etc.[22] We model $\mathbf{R}$ as a diagonal matrix where $\sigma^2_{\mathbf{R}}$ is the sum of several components scaled by parameters optimized using Maximum Likelihood.[19]

$$\sigma^2_{\mathbf{R}} = (\mathbf{y_{twr,unc}}^2 + (\mathbf{H}_{WRF}\boldsymbol{s_{unit}} - \mathbf{H}_{ERA5}\boldsymbol{s_{unit}})^2 + \left(\mathbf{BG}_{\boldsymbol{gb,CAMS}} - \mathbf{BG}_{\boldsymbol{gb,CTCH4}}\right)^2)\theta_1 + \mathbf{I}\theta_2 \qquad \text{Eq. S-11}$$

Where $\mathbf{y_{twr,unc}}^2$, is the sub-hourly standard deviation of the observations, $(\mathbf{H}_{WRF}\boldsymbol{s_{unit}} - \mathbf{H}_{ERA}\boldsymbol{s_{unit}})^2$ is the square of the difference of a unit flux prescribed in all of the d01 regional domain convolved by the $\mathbf{H}_{WRF}$ and $\mathbf{H}_{ERA}$ (which is a proxy for transport error), $\left(\mathbf{BG}_{\boldsymbol{gb,CAMS}} - \mathbf{BG}_{\boldsymbol{gb,CTCH4}}\right)^2$ is the square difference of the CAMS17rs1 and CTCH4 backgrounds for each observation (which is a proxy for background error), and $\mathbf{I}$ is an identity matrix that allows us to capture other gaussian errors that are not attributed to measurement, transport, and background errors – such as those associated with misrepresenting wetland emissions.

$\mathbf{Q}$ matrix is the prior error covariance matrix, which characterizes the square of the residuals between $\boldsymbol{\lambda_p}$ and $\hat{\boldsymbol{\lambda}}$. We model $\mathbf{Q}$ as:

$$\sigma^2_{\mathbf{Q}} = \begin{bmatrix} \theta_3 & 0 & 0 \\ 0 & \theta_3 & 0 \\ 0 & 0 & 3 \end{bmatrix} \qquad \text{Eq. S-12}$$

We estimate all parameter, aka $\theta_1, \theta_2, \theta_3$ using Maximum Likelihood.[23] Note that $\theta_3 > 0$. We assigned 3 as the variance on the λ_3 because if assigned it significantly impacted the $\chi^2{}_{Red}$.

SI-6: Inversion Filtering, Estimation Domain Evaluation, Number of Estimates in Monthly Means, Inversion Metrics, and Estimated Scaling Factors

Inversion Filtering:

We filter our inversion estimates by not including estimates that do not pass the following metrics.

To ensure that there is enough data constraint in each the 8-day inversion period, we only estimate scaling factors for inversions where there are at least 10 observations. We also filter the inversion results using ${\chi^2}_{\mathbf{Q}} < 6$, where ${\chi^2}_{\mathbf{Q}}$ quantifies the agreement between the posterior flux estimates and the prior, weighted by the prior covariance matrix **Q** (Eq. S-13). This threshold ensures that the inversion results remain consistent with prior knowledge while incorporating observational constraints. The threshold is chosen based on the expected distribution of ${\chi^2}_{\mathbf{Q}}$ for a well-defined prior and approximately 2-3 degrees of freedom, reflecting the aggregated nature of the flux estimates. Filtering helps to exclude poorly constrained or statistically inconsistent estimates, improving the robustness of the results. Note with 10 observations and three scaling factor estimates, the degrees of freedom are approximately 7.

$${\chi^2}_{\mathbf{Q}} = (\boldsymbol{\lambda}_{\mathbf{p}} - \hat{\boldsymbol{\lambda}})^T \mathbf{Q}^{-1} (\boldsymbol{\lambda}_{\mathbf{p}} - \hat{\boldsymbol{\lambda}}) \quad \text{Eq. S-13}$$

Number of Estimates in Monthly Means:

Inversion Metrics:

The following metrics are used to assess the inversion.

Our estimated enhancements are:

$$\hat{y} = \widehat{\boldsymbol{\lambda}_1}\boldsymbol{Hs}_{p,in} + \widehat{\boldsymbol{\lambda}_2}\boldsymbol{Hs}_{p,out} + \mathbf{Hs_{wet}} - \widehat{\boldsymbol{\lambda}_3}(\mathbf{BG}_{gb} - 10.22) \quad \text{Eq. S-14}$$

And our modelled (prior, referred to ps) enhancements are:

$$y_{ps} = \boldsymbol{Hs}_{p,in} + \boldsymbol{Hs}_{p,out} + \boldsymbol{BG}_{gb} - 10.22 \quad \text{Eq. S-15}$$

The biases in Fig S-X (Where N is the number of observations) are defined as:

$$Bias\ (est) = \frac{1}{N}\sum_{i=1}^{N}(y_{twr,i} - \hat{y}_i) \quad \text{Eq. S-16}$$

$$Bias\ (ps) = \frac{1}{N}\sum_{i=1}^{N}(y_{twr,i} - y_{ps,i}) \quad \text{Eq. S-17}$$

Where N is the number of observations.

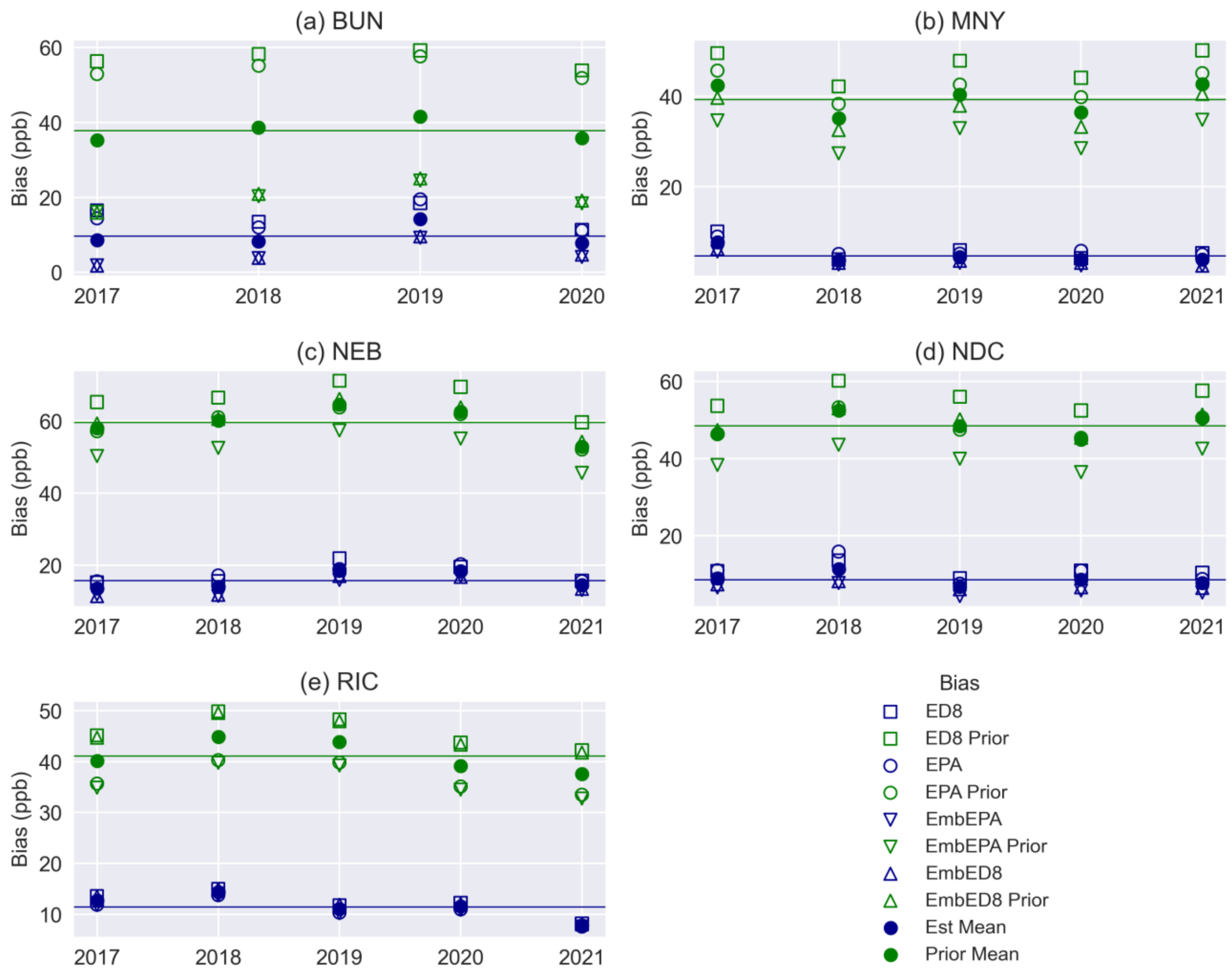

Figure S17: Biases of $\hat{y}$ (blue) and y_{ps} (green) for each of the inventory ensemble members (symbols) and for each year. Note for each symbol, the inventory estimates associated with WRF and ERA5 are averaged. The mean estimated biases of $\hat{y}$ (Est Mean; blue circles) and the mean y_{ps} (Prior mean; green circle and lines) are also shown across years.

The reduced chi-square Yadav et al.[24] compares the discrepancy between observed and modeled concentrations against the uncertainties in the data and prior emissions. A chi-squared value close to one suggests that the model's uncertainty estimates are likely consistent with the posterior uncertainties. A chi-squared value much greater than one suggests that uncertainties in **R** may be underestimated, leading to an overconfident posterior. Conversely, a chi-square value much less than one suggests that uncertainties in **R** may be overestimated, leading to an overly conservative posterior. The equation is:

$$\chi^2{}_{Red} = \frac{\left(y_{twr} - \widehat{\lambda_1}\mathbf{Hs}_{p,in} - \widehat{\lambda_2}\mathbf{Hs}_{p,out} - \mathbf{Hs}_{\mathbf{wet}} - \widehat{\lambda_3}(\mathbf{BG}_{gb} - \mathbf{BG}_{adj})\right)^{\mathbf{T}} \mathbf{R}^{-1}\left(y_{twr} - \widehat{\lambda_1}\mathbf{Hs}_{p,in} - \widehat{\lambda_2}\mathbf{Hs}_{p,out} - \mathbf{Hs}_{\mathbf{wet}} - \widehat{\lambda_3}(\mathbf{BG}_{gb} - \mathbf{BG}_{adj})\right) + (\boldsymbol{\lambda}_{\mathbf{p}} - \hat{\boldsymbol{\lambda}})^{T}\mathbf{Q}^{-1}(\boldsymbol{\lambda}_{\mathbf{p}} - \hat{\boldsymbol{\lambda}})}{\nu}$$

Eq. S18

Where ν is the number of estimated parameters, or three associated with each of our scaling factors.

Root Mean Square Error (RMSE) quantifies the overall model error. Our equations are:

Root Mean Square Error (RMSE) quantifies the overall model error. Our equations are:

$$RMSE_{\hat{y}} = \sqrt{\frac{1}{N}\sum_{i=1}^{N}\left(y_{twr,i} - \hat{y}_i\right)^2} \quad \text{Eq. S-19}$$

$$RMSE_{yps} = \sqrt{\frac{1}{N}\sum_{i=1}^{N}\left(y_{twr,i} - y_{ps,i}\right)^2} \quad \text{Eq. S-20}$$

The coefficient of determination (R^2), which represents the proportion of the variance in $\boldsymbol{y_{twr}}$ that is explained by $\hat{\mathbf{y}}$ or $\mathbf{y_{ps}}$. Our equations are:

$$R^2{}_{\hat{y}} = 1 - \frac{\frac{1}{N}\sum_{i=1}^{N}\left(y_{twr,i} - y_{ps,i}\right)^2}{\frac{1}{N}\sum_{i=1}^{N}\left(y_{twr,i} - \bar{y}\right)^2} \quad \text{Eq. S-21}$$

$$R^2{}_{yps} = 1 - \frac{\frac{1}{N}\sum_{i=1}^{N}\left(y_{twr,i} - y_{ps,i}\right)^2}{\frac{1}{N}\sum_{i=1}^{N}\left(y_{twr,i} - \bar{y}\right)^2} \quad \text{Eq. S-22}$$

Note, a negative R^2 typically indicates that the model performs worse than a baseline model that simply predicts the mean of the observed data. While R^2 is commonly expected to be between 0 and 1, it can become negative in certain situations.

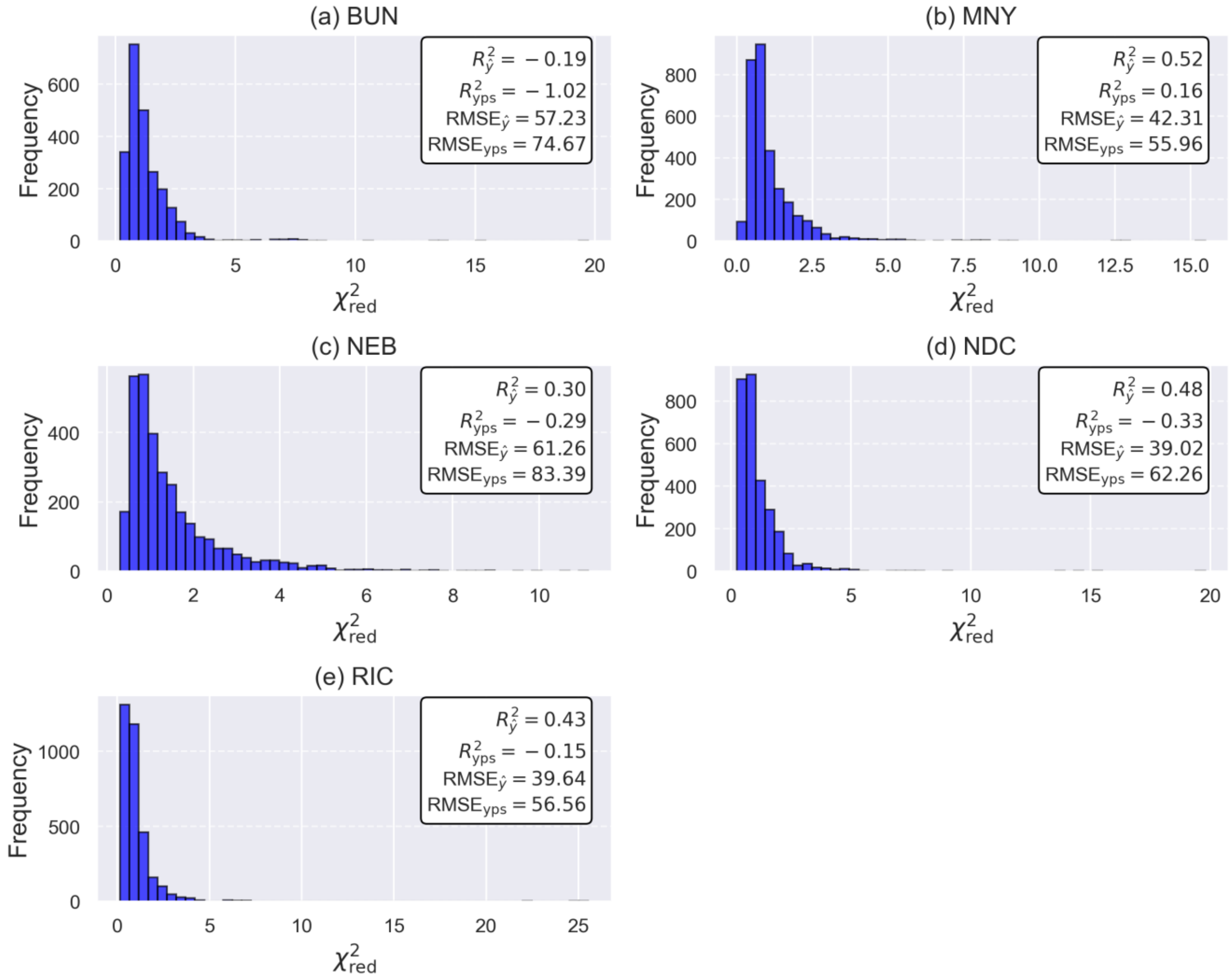

Figure S18: ${\chi^2}_{Red}$ values for all inversions over all years. These values, which are largely centered on one, are positively skewed which suggests that overall, the uncertainties associated with the inversion are largely consistent with those in **R** and **Q**. However, the skewness suggests that are some instances where the model performs worse than expected for some times. This is expected given that we are using a linear model to non-linear data, especially if there are outliers. Given that the other metrics are reasonable, we keep these larger ${\chi^2}_{Red}$ within our results. We did check removing these estimates, although they did not significantly impact the results or other metrics but did limit the data used for our estimates. Thus, to ensure a more robust result, we included these estimates.

The posterior covariance matrix, $\mathbf{V}_{\hat{\lambda}}$, quantifies the variances and covariances between different scaling parameters. Element $\mathbf{V}_{\hat{\lambda}i,,j}$ represents the covariance between estimates i and j, while the diagonal elements $\boldsymbol{i,i}$ represent the posterior variances. We convert the covariance matrix $\mathbf{V}_{\hat{\lambda}}$, into the corresponding correlation matrix $Rho\mathbf{V}_{\hat{\lambda}}$ (Eq. S-24) to investigate how independent the estimates are from one another – especially $\hat{\boldsymbol{\lambda}}_1$and $\hat{\boldsymbol{\lambda}}_2$.

$$Rho\mathbf{V}_{\hat{\lambda}} = \frac{\mathbf{V}_{\hat{\lambda},i,j}}{\sqrt{\mathbf{V}_{\hat{\lambda},i,i}.\mathbf{V}_{\hat{\lambda},j,j}.}} \quad \text{Eq. S-22}$$

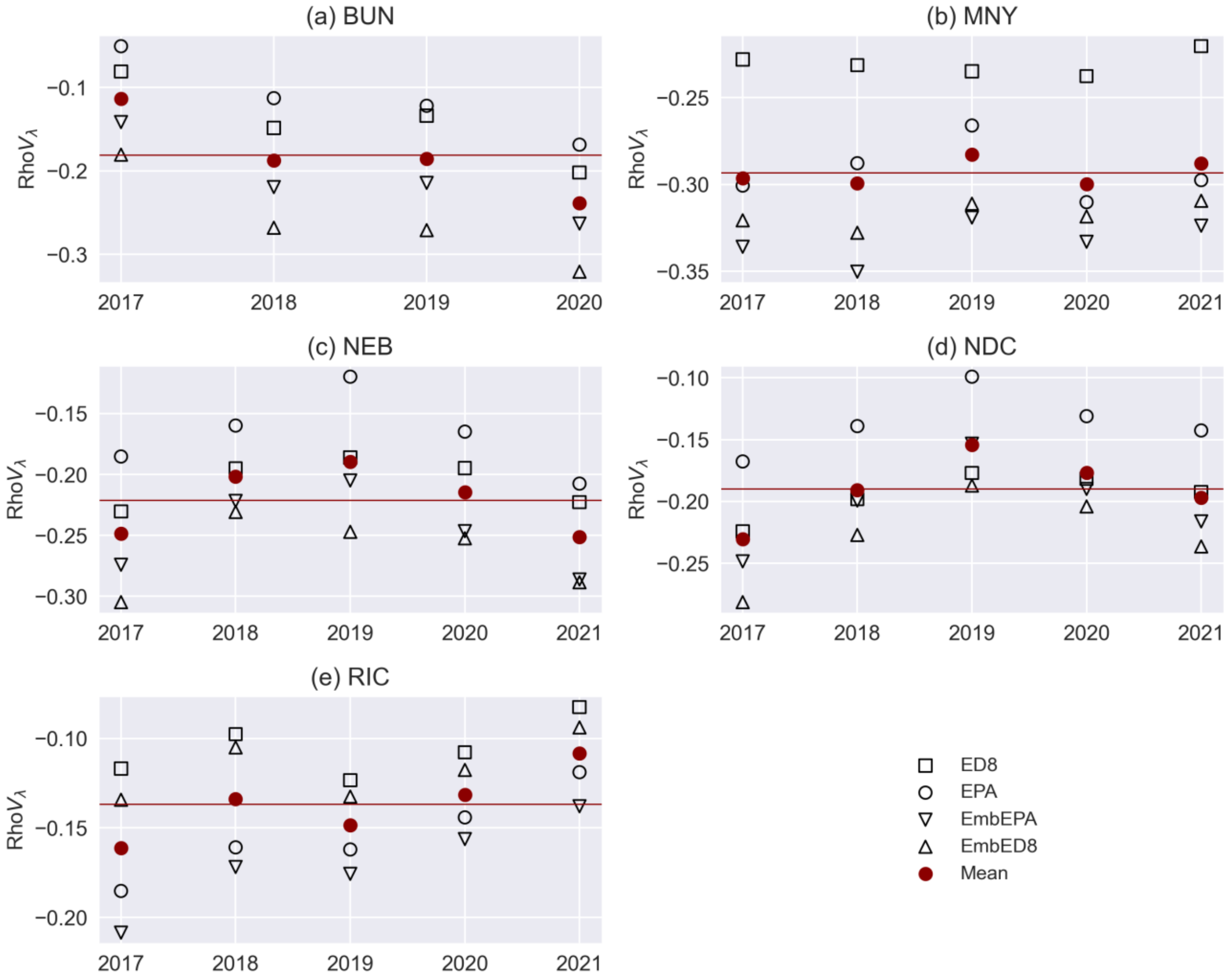

Figure S19: $Rho\mathbf{V}_{\hat{\lambda}}$ values for all the emission inventories across years. Note for each black symbol, the inventory estimates associated with WRF and ERA5 are averaged. The mean $Rho\mathbf{V}_{\hat{\lambda}}$ across all inventories and metrologies are shown in red with the red solid line being the average across years.

Estimation Domain Evaluation:

As noted in the main text, we also compare the variability in modeled CH_4 enhancements (see Eq-2, main text) arising from emissions (see Section 2.3 text and SI-3) within and outside the estimation domain. To do this, we have two components (1) modelled enhancements from the 50 km estimation domain (zeroed outside in the regional d01 domain, Fig-2) ($\mathbf{Hs}_{p,in}$), and (2) modelled enhancements from emissions in the regional d01 domain (zeroed within the 50 km local estimation domain, Fig-2) ($\mathbf{Hs}_{p,out}$). We look at the standard deviations of the modelled enhancements within an 8-day estimation period and divide these by the mean of the modelled enhancements (Table-S3, Eq-S14).

$$Relative\ Difference = \left(\frac{\sigma_{\mathbf{Hsin}}}{\mathbf{Hsin}} - \frac{\sigma_{\mathbf{Hsout}}}{\mathbf{Hsout}}\right) / \frac{\sigma_{\mathbf{Hsin}}}{\mathbf{Hsin}} \qquad \text{Eq. S-23}$$

Table-S3: Relative difference (Eq. S-14) in the standard deviations of the inside $\mathbf{Hs}_{p,in}$ and outside $\mathbf{Hs}_{p,out}$ across an 8-day estimation period normalized by total mean $\mathbf{Hs}_{p,in}$ and $\mathbf{Hs}_{p,out}$ respectively for each of the inventory ensemble members. **H** was generated using either WRF or ERA5, as indicated by the first column.

Met Variable	Tower	ED8	EPA	EmbED8	EmbEPA
WRF	BUN	25%	28%	24%	23%
WRF	MNY	12%	33%	37%	40%
WRF	NEB	48%	41%	37%	36%
WRF	NDC	51%	30%	35%	31%
WRF	RIC	44%	46%	45%	47%
ERA5	BUN	56%	62%	49%	57%
ERA5	MNY	8%	40%	35%	44%
ERA5	NEB	67%	59%	56%	57%
ERA5	NDC	32%	21%	24%	25%
ERA5	RIC	48%	48%	49%	49%

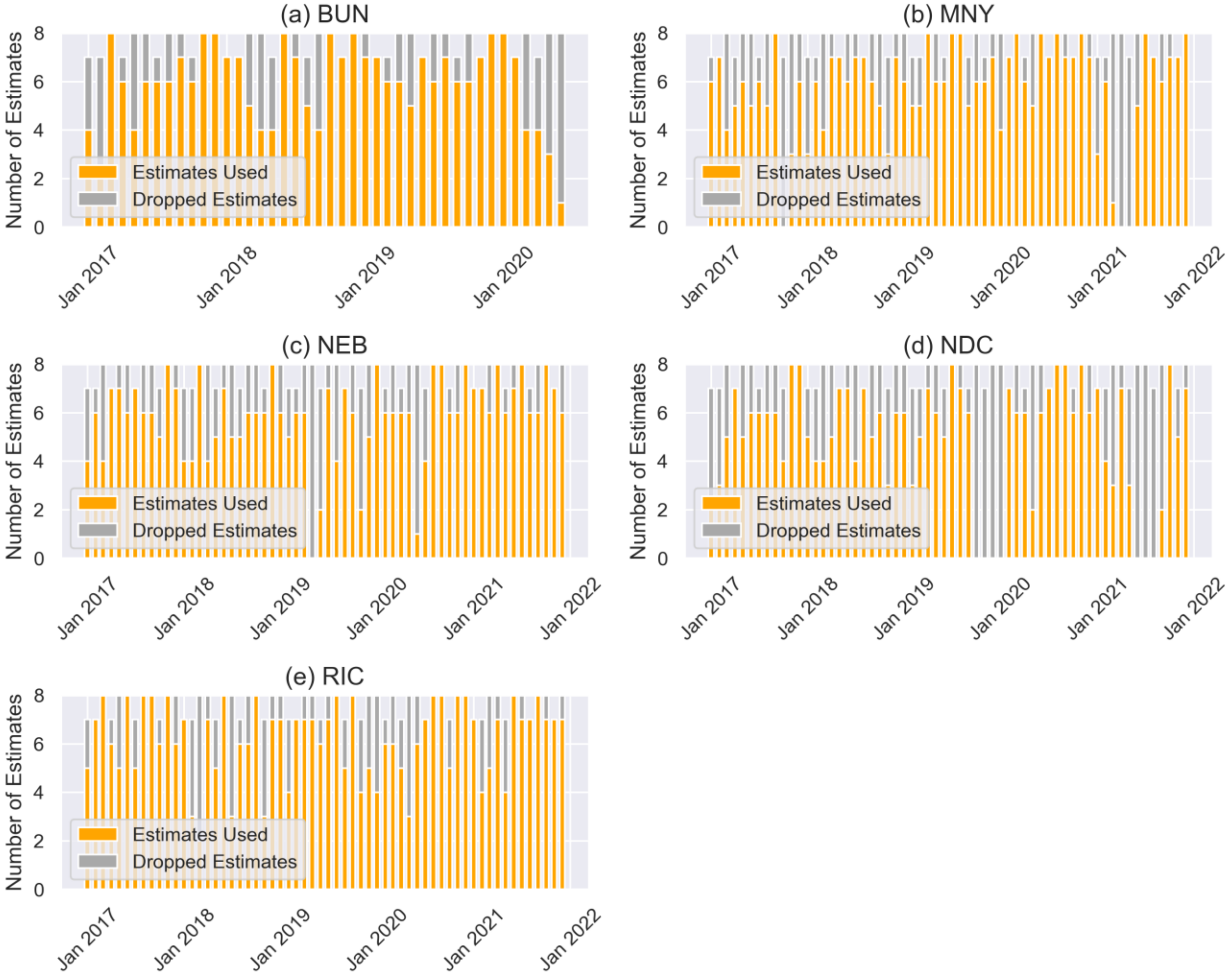

Figure S20: The number of valid estimates (e.g., those that have more than 10 measurements, etc. in each 8-day period) are shown in yellow, while potential estimation time periods that were filtered and not included (dropped

estimates) are shown in grey. We only show months in Fig-4 that have three or more estimates in each month to ensure that the monthly estimates are not biased.

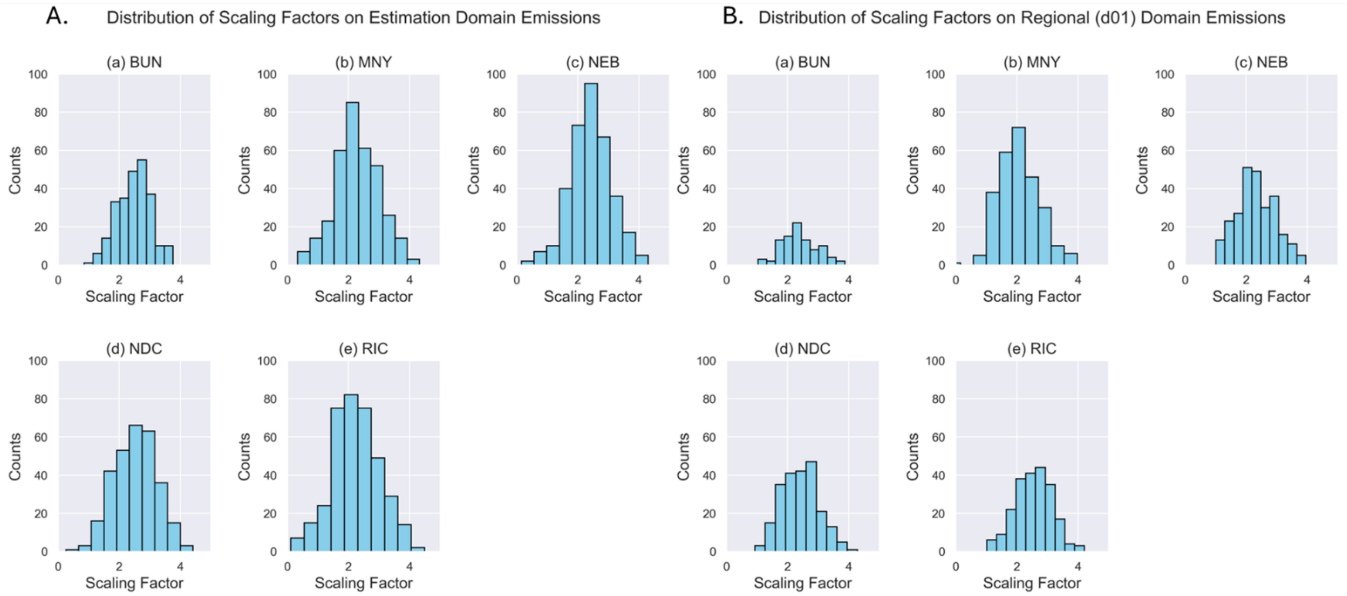

Figure S21: Histograms of estimated scaling factors on (A) estimation domain emissions ($\boldsymbol{s_{p,in}}$) and (B) Regional (d01) Domain emissions ($\boldsymbol{s_{p,out}}$) for each of the five tower locations.

SI-7: Characteristic Analysis – Data

Data were compiled from a variety of sources, as detailed in Table-S3. Natural gas deliveries and the lengths of pipeline mains and services were collected from reports by Local Distribution Companies (LDCs) available in the EPA's Greenhouse Gas Reporting Program (GHGRP) FLIGHT tool[25] To spatially disaggregate LDC data, road networks from OpenStreetMap[26] were utilized: primary and secondary roads were used to represent mains, while secondary and tertiary roads were used for services. These spatially disaggregated data were aggregated to 0.1 degree grid cells within the 50 km local estimation domain to maintain consistency across datasets.

Landfill emissions were estimated using the Landfill Methane Outreach Program (LMOP) database[27], with the Waste Acceptance Rate serving as a proxy for emissions. For wastewater treatment emissions, municipal wastewater treatment plants were associated with receiving population, calculated from the EPA's Clean Water Needs 2024 Survey[28]. Population data were obtained at the census tract level from the U.S. Census Bureau[29] and aggregated to the 0.1 degree grid cells to align with the spatial resolution of other datasets. Census data were further used to determine the age of housing and the number of households heating with natural gas, which were similarly aggregated to the 0.1 degree scale (see Fig-S17 for examples of the spatial distribution of these Census data).

Commercial, residential, and industrial building volumes were calculated using the Homeland Infrastructure Foundation-Level Data (HIFLD) Structures database.[30] The building volume was estimated as the product of the building footprint area (in square meters) and the building height. In densely populated areas, the reported height was used when available, while in rural areas, the building height was approximated as 1.5 stories. Stationary source emissions were compiled directly from the EPA's GHGRP

FLIGHT tool, providing a comprehensive overview of large point sources of emissions within the estimation domain.

Table-S4: A list of the characteristic data, their values, and sources for the five different estimation domains used in this study

Tower	BUN	MNY	NEB	NDC	RIC	Source
Mains and Services (km)	40,092	44,784	25,504	28,271	15,637	USGHGRP
Waste Acceptance Rate (Mmtonne)	476	20	116	1,657	4,222	USGHGRP
Residential NG Deilveries (MMscf)	65,037	403,182	51,873	101,171	13,432	USGHGRP
Commercial NG Deliveries (MMscf)	77,084	278,260	42,249	91,039	12,765	USGHGRP
Industrial NG Deliveries (MMscf)	26,056	59,283	14,843	15,333	24,259	USGHGRP
Receiving Population Municipal Waste Water Treatment Plant (WWTP)	6,989,346	18,557,935	5,034,427	6,550,772	1,046,929	CNWS
Point Sources (tonnes)	63	-	240	489	-	USGHGRP
Population	6,731,206	14,251,756	3,810,458	6,567,237	1,267,496	Census American Community Survey 5-year
Residential Housing Units that heat with NG	1,489,437	3,557,738	728,831	1,278,198	147,710	Census American Community Survey 5-year
Median Age of Residential Housing	92	62	45	46	37	Census American Community Survey 5-year
Residential Volume (km3)	1.28	1.92	0.87	1.57	0.32	HFLID Structures Database
Commercial Volume (km2)	0.18	0.46	0.16	0.38	0.05	HFLID Structures Database
Industrial Volume (km2)	0.09	0.35	0.17	0.17	0.03	HFLID Structures Database

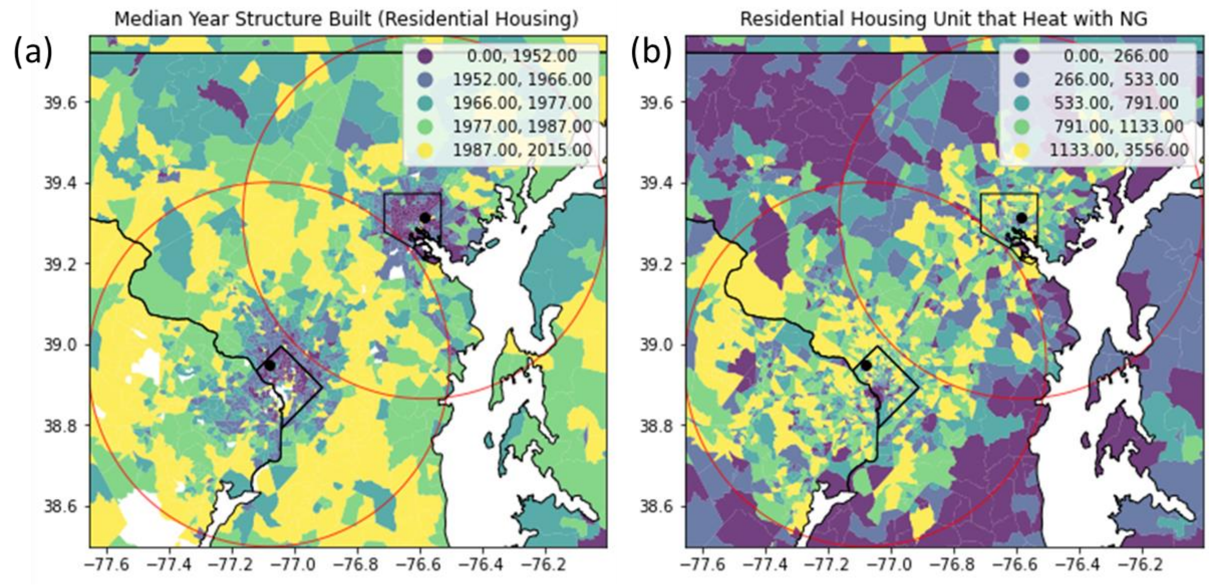

Figure S22: Examples of the spatial distribution of Census tract level data for estimation areas around NEB (Baltimore) and NDC (Washington DC).

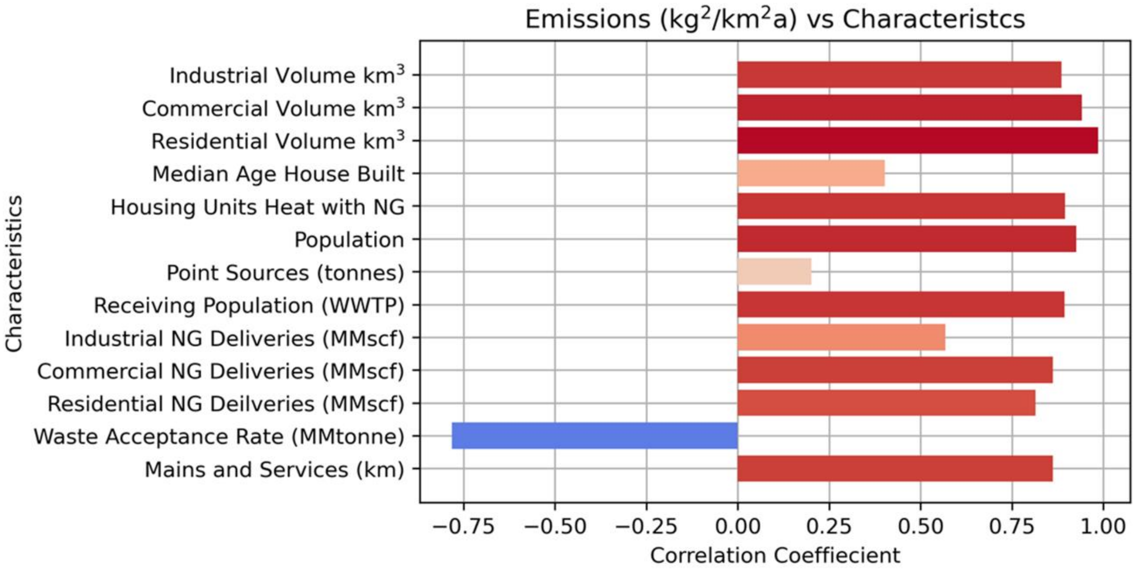

Figure S23: Correlation coefficients between each characteristic dataset and emissions.

References

[1] Gourdji SM, Karion A, Lopez-Coto I, Ghosh S, Mueller KL, Zhou Y, Williams CA, Baker IT, Haynes KD, Whetstone JR. A Modified Vegetation Photosynthesis and Respiration Model (VPRM) for the Eastern USA and Canada, Evaluated With Comparison to Atmospheric Observations and Other Biospheric Models. J Geophys Res Biogeosciences 2022;127:e2021JG006290. https://doi.org/10.1029/2021JG006290.

[2] Gourdji SM, Mueller KL, Yadav V, Huntzinger DN, Andrews AE, Trudeau M, Petron G, Nehrkorn T, Eluszkiewicz J, Henderson J, Wen D, Lin J, Fischer M, Sweeney C, Michalak AM. North American CO_2

exchange: inter-comparison of modeled estimates with results from a fine-scale atmospheric inversion. Biogeosciences 2012;9:457–75. https://doi.org/10.5194/bg-9-457-2012.
[3] Methane Emissions Show Recent Decline but Strong Seasonality in Two US Northeastern Cities | Environmental Science & Technology n.d. https://pubs.acs.org/doi/10.1021/acs.est.3c05050 (accessed January 20, 2025).
[4] Lin JC, Gerbig C, Wofsy SC, Andrews AE, Daube BC, Davis KJ, Grainger CA. A near-field tool for simulating the upstream influence of atmospheric observations: The Stochastic Time-Inverted Lagrangian Transport (STILT) model. J Geophys Res Atmospheres 2003;108. https://doi.org/10.1029/2002JD003161.
[5] Karion A, Ghosh S, Lopez-Coto I, Mueller K, Gourdji S, Pitt J, Whetstone J. Methane Emissions Show Recent Decline but Strong Seasonality in Two US Northeastern Cities. Environ Sci Technol 2023;57:19565–74. https://doi.org/10.1021/acs.est.3c05050.
[6] Hersbach H, Bell B, Berrisford P, Hirahara S, Horányi A, Muñoz-Sabater J, Nicolas J, Peubey C, Radu R, Schepers D, Simmons A, Soci C, Abdalla S, Abellan X, Balsamo G, Bechtold P, Biavati G, Bidlot J, Bonavita M, De Chiara G, Dahlgren P, Dee D, Diamantakis M, Dragani R, Flemming J, Forbes R, Fuentes M, Geer A, Haimberger L, Healy S, Hogan RJ, Hólm E, Janisková M, Keeley S, Laloyaux P, Lopez P, Lupu C, Radnoti G, de Rosnay P, Rozum I, Vamborg F, Villaume S, Thépaut J-N. The ERA5 global reanalysis. Q J R Meteorol Soc 2020;146:1999–2049. https://doi.org/10.1002/qj.3803.
[7] Skamarock WC, Klemp JB, Dudhia J, Gill DO, Liu Z, Berner J, Wang W, Powers JG, Duda MG, Barker DM, Huang X-Y. A Description of the Advanced Research WRF Model Version 4. UCAR/NCAR; 2019. https://doi.org/10.5065/1DFH-6P97.
[8] Pitt JR, Lopez-Coto I, Karion A, Hajny KD, Tomlin J, Kaeser R, Jayarathne T, Stirm BH, Floerchinger CR, Loughner CP, Commane R, Gately CK, Hutyra LR, Gurney KR, Roest GS, Liang J, Gourdji S, Mueller KL, Whetstone JR, Shepson PB. Underestimation of Thermogenic Methane Emissions in New York City. Environ Sci Technol 2024;58:9147–57. https://doi.org/10.1021/acs.est.3c10307.
[9] Sargent MR, Floerchinger C, McKain K, Budney J, Gottlieb EW, Hutyra LR, Rudek J, Wofsy SC. Majority of US urban natural gas emissions unaccounted for in inventories. Proc Natl Acad Sci 2021;118:e2105804118. https://doi.org/10.1073/pnas.2105804118.
[10] Mead GJ, Waxman EM, Bon D, Herman DI, Baumann E, Giorgetta FR, Friedlein JT, Ycas G, Newbury NR, Coddington I, Cossel KC. Open-path dual-comb spectroscopy of methane and VOC emissions from an unconventional oil well development in Northern Colorado. Front Chem 2023;11. https://doi.org/10.3389/fchem.2023.1202255.
[11] Agusti-Panareda A, Diamantakis M, Bayona V, Klappenbach F, Butz A. Improving the inter-hemispheric gradient of total column atmospheric CO_2 and CH_4 in simulations with the ECMWF semi-Lagrangian atmospheric global model. Geosci Model Dev 2017;10:1–18. https://doi.org/10.5194/gmd-10-1-2017.
[12] Oh Y, Bruhwiler L, Lan X, Basu S, Schuldt K, Thoning K, Michel SE, Clark R, Miller JB, Sherwood O, Etiope G, Crippa M, Liu L, Zhuang Q, Randerson J, van der Werf G, Aalto T, Amendola S, Andra SC, Andrade M, Nguyen NA, Aoki S, Apadula F, Arifin IB, Arnold S, Arshinov M, Baier B, Bergamaschi P, Biermann T, Biraud SC, Blanc P-E, Brailsford G, Chen H, Colomb A, Couret C, Cristofanelli P, Cuevas E, Chmura L, Delmotte M, Emmenegger L, Esenzhanova G, Fujita R, Gatti L, Guerette E-A, Haszpra L, Heliasz M, Hermansen O, Holst J, Di lorio T, Jordan A, Jennifer M-W, Karion A, Kawasaki T, Kazan V, Keronen P, Kim S-Y, Kneuer T, Kominkova K, Kozlova E, Krummel P, Kubistin D, Labuschagne C, Langenfelds R, Laurent O, Laurila T, Lee H, Lehner I, Leuenberger M, Lindauer M, Lopez M, Mahdi R, Mammarella I, Manca G, Marek MV, Mazière MD, McKain K, Meinhardt F, Miller CE, Mölder M, Moncrieff J, Moosen H, Moreno C, Morimoto S, Myhre CL, Nahas AC, Necki J, Nichol S, O’Doherty S, Paramonova N, Piacentino S, Pichon JM, Plass-Dülmer C, Ramonet M, Ries L, di Sarra AG, Sasakawa M, Say D, Schaefer H, Scheeren B, Schmidt M, Schumacher M, Sha MK, Shepson P, Smale D, Smith

PD, Steinbacher M, Sweeney C, Takatsuji S, Torres G, Tørseth K, Trisolino P, Turnbull J, Uhse K, Umezawa T, Vermeulen A, Vimont I, Vitkova G, Wang H-J (Ray), Worthy D, Xueref-Remy I. CarbonTracker CH4 2023 2023. https://doi.org/10.25925/40JT-QD67.
[13] Janssens-Maenhout G. EDGARv4.2 Emission Maps 2011.
[14] Janssens-Maenhout G, Crippa M, Guizzardi D, Muntean M, Schaaf E, Dentener F, Bergamaschi P, Pagliari V, Olivier JGJ, Peters JAHW, van Aardenne JA, Monni S, Doering U, Petrescu AMR, Solazzo E, Oreggioni GD. EDGAR v4.3.2 Global Atlas of the three major greenhouse gas emissions for the period 1970–2012. Earth Syst Sci Data 2019;11:959–1002. https://doi.org/10.5194/essd-11-959-2019.
[15] Ferrario FM, Crippa M, Guizzardi D, Muntean M, Schaaf E, Vullo EL, Solazzo E, Olivier J, Vignati E. EDGAR v5.0 Greenhouse Gas Emissions 2019.
[16] Crippa M, Solazzo E, Huang G, Guizzardi D, Koffi E, Muntean M, Schieberle C, Friedrich R, Janssens-Maenhout G. High resolution temporal profiles in the Emissions Database for Global Atmospheric Research. Sci Data 2020;7:121. https://doi.org/10.1038/s41597-020-0462-2.
[17] Kenneth N. Schuldt, Tuula Aalto, Arlyn Andrews, Shuji Aoki, Jgor Arduini, Bianca Baier, Peter Bergamaschi, Tobias Biermann, Sebastien C. Biraud, Harald Boenisch, Gordon Brailsford, Huilin Chen, Aurelie Colomb, Sébastien Conil, Paolo Cristofanelli, Emilio Cuevas, Bruce Daube, Kenneth Davis, Martine De Mazière, Marc Delmotte, Ankur Desai, Joshua P. DiGangi, Ed Dlugokencky, James W. Elkins, Lukas Emmenegger, Marc L. Fischer, Luciana V. Gatti, Torsten Gehrlein, Christoph Gerbig, Emanuel Gloor, Daisuke Goto, László Haszpra, Juha Hatakka, Martin Heimann, Michal Heliasz, Ove Hermanssen, Eric Hintsa, Jutta Holst, Viktor Ivakhov, Dan Jaffe, Warren Joubert, Hui-Yun Kang, Anna Karion, Victor Kazan, Petri Keronen, Mi-Young Ko, Katerina Kominkova, Eric Kort, Elena Kozlova, Paul Krummel, Dagmar Kubistin, Casper Labuschagne, Ray Langenfelds, Olivier Laurent, Tuomas Laurila, Thomas Lauvaux, John Lee, Haeyoung Lee, Choong-Hoon Lee, Irene Lehner, Reimo Leppert, Markus Leuenberger, Matthias Lindauer, Zoe Loh, Morgan Lopez, Toshinobu Machida, Ivan Mammarella, Giovanni Manca, Michal V. Marek, Melissa Y. Martin, Hidekazu Matsueda, Kathryn McKain, Natasha Miles, Charles E. Miller, John B. Miller, Fred Moore, Shinji Morimoto, David Munro, Cathrine L. Myhre, Meelis Mölder, Jennifer Müller-Williams, Sylvia Nichol, Yosuke Niwa, Simon O\'Doherty, Florian Obersteiner, Salvatore Piacentino, Jean M. Pichon, Jasna Pittman, Christian Plass-Duelmer, Michel Ramonet, Scott Richardson, Pedro P. Rivas, Kazuyuki Saito, Greg Santoni, Motoki Sasakawa, Bert Scheeren, Tanja Schuck, Marcus Schumacher, Thomas Seifert, Mahesh K. Sha, Paul Shepson, Christopher D. Sloop, Paul Smith, Martin Steinbacher, Britton Stephens, Colm Sweeney, Helder Timas, Margaret Torn, Pamela Trisolino, Jocelyn Turnbull, Kjetil Tørseth, Brian Viner, Gabriela Vitkova, Andrew Watson, Steve Wofsy, Justin Worsey, Doug Worthy, Andreas Zahn, Alcide G. di Sarra. Multi-laboratory compilation of atmospheric methane data for the period 1983-2020; obspack_ch4_1_GLOBALVIEWplus_v4.0_2021-10-14 2021. https://doi.org/10.25925/20211001.
[18] Mueller KL, Yadav V, Curtis PS, Vogel C, Michalak AM. Attributing the variability of eddy-covariance CO2 flux measurements across temporal scales using geostatistical regression for a mixed northern hardwood forest. Glob Biogeochem Cycles 2010;24. https://doi.org/10.1029/2009GB003642.
[19] Michalak AM, Hirsch A, Bruhwiler L, Gurney KR, Peters W, Tans PP. Maximum likelihood estimation of covariance parameters for Bayesian atmospheric trace gas surface flux inversions. J Geophys Res Atmospheres 2005;110. https://doi.org/10.1029/2005JD005970.
[20] Yadav V, Ghosh S, Miller CE. Metrics for evaluating the quality in linear atmospheric inverse problems: a case study of a trace gas inversion. Geosci Model Dev 2023;16:5219–36. https://doi.org/10.5194/gmd-16-5219-2023.
[21] Richardson SJ, Miles NL, Davis KJ, Lauvaux T, Martins DK, Turnbull JC, McKain K, Sweeney C, Cambaliza MOL. Tower measurement network of in-situ CO2, CH4, and CO in support of the

Indianapolis FLUX (INFLUX) Experiment. Elem Sci Anthr 2017;5:59. https://doi.org/10.1525/elementa.140.
[22] Engelen RJ, Denning AS, Gurney KR. On error estimation in atmospheric CO2 inversions. J Geophys Res Atmospheres 2002;107:ACL 10-1-ACL 10-13. https://doi.org/10.1029/2002JD002195.
[23] Michalak AM, Hirsch A, Bruhwiler L, Gurney KR, Peters W, Tans PP. Maximum likelihood estimation of covariance parameters for Bayesian atmospheric trace gas surface flux inversions. J Geophys Res Atmospheres 2005;110. https://doi.org/10.1029/2005JD005970.
[24] Yadav V, Duren R, Mueller K, Verhulst KR, Nehrkorn T, Kim J, Weiss RF, Keeling R, Sander S, Fischer ML, Newman S, Falk M, Kuwayama T, Hopkins F, Rafiq T, Whetstone J, Miller C. Spatio-temporally Resolved Methane Fluxes From the Los Angeles Megacity. J Geophys Res Atmospheres 2019;124:5131–48. https://doi.org/10.1029/2018JD030062.
[25] EPA Facility Level GHG Emissions Data n.d. https://ghgdata.epa.gov/ghgp/main.do (accessed January 28, 2025).
[26] OpenStreetMap. OpenStreetMap n.d. https://www.openstreetmap.org/ (accessed January 28, 2025).
[27] US EPA O. LMOP Landfill and Project Database 2016. https://www.epa.gov/lmop/lmop-landfill-and-project-database (accessed January 28, 2025).
[28] US EPA O. Clean Watersheds Needs Survey 2015. https://www.epa.gov/cwns (accessed January 28, 2025).
[29] Bureau UC. Data. CensusGov n.d. https://www.census.gov/data (accessed January 28, 2025).
[30] HIFLD n.d. https://hifld-geoplatform.hub.arcgis.com/ (accessed January 28, 2025).
